# Insights and approaches using deep learning to classify wildlife

**DOI:** 10.1038/s41598-019-44565-w

**Published:** 2019-05-31

**Authors:** Zhongqi Miao, Kaitlyn M. Gaynor, Jiayun Wang, Ziwei Liu, Oliver Muellerklein, Mohammad Sadegh Norouzzadeh, Alex McInturff, Rauri C. K. Bowie, Ran Nathan, Stella X. Yu, Wayne M. Getz

**Affiliations:** 1 Dept. Env. Sci., Pol. & Manag., UC Berkeley, Berkeley, CA United States; 2 International Comp. Sci. Inst., UC Berkeley, 1947 Center St, Berkeley, CA United States; 3 Vision Sci. Grad. Group, UC Berkeley, Berkeley, CA United States; 4 Dept. Comp. Sci., U. Wyoming, Laramie, WY United States; 5 Dept. Integr. Biol. & Museum of Vertebrate Zoology, UC Berkeley, Berkeley, CA United States; 6 Dept. EEB, Alexander Silberman Inst. Life Sci., Hebrew U. Jerusalem, Givat Ram, Jerusalem, Israel; 7 Sch. Math. Sci., Univ. KwaZulu-Natal, Durban, South Africa

**Keywords:** Image processing, Population dynamics

## Abstract

The implementation of intelligent software to identify and classify objects and individuals in visual fields is a technology of growing importance to operatives in many fields, including wildlife conservation and management. To non-experts, the methods can be abstruse and the results mystifying. Here, in the context of applying cutting edge methods to classify wildlife species from camera-trap data, we shed light on the methods themselves and types of features these methods extract to make efficient identifications and reliable classifications. The current state of the art is to employ convolutional neural networks (CNN) encoded within deep-learning algorithms. We outline these methods and present results obtained in training a CNN to classify 20 African wildlife species with an overall accuracy of 87.5% from a dataset containing 111,467 images. We demonstrate the application of a gradient-weighted class-activation-mapping (Grad-CAM) procedure to extract the most salient pixels in the final convolution layer. We show that these pixels highlight features in particular images that in some cases are similar to those used to train humans to identify these species. Further, we used mutual information methods to identify the neurons in the final convolution layer that consistently respond most strongly across a set of images of one particular species. We then interpret the features in the image where the strongest responses occur, and present dataset biases that were revealed by these extracted features. We also used hierarchical clustering of feature vectors (i.e., the state of the final fully-connected layer in the CNN) associated with each image to produce a visual similarity dendrogram of identified species. Finally, we evaluated the relative unfamiliarity of images that were not part of the training set when these images were one of the 20 species “known” to our CNN in contrast to images of the species that were “unknown” to our CNN.

## Introduction

Collecting animal imagery data with motion sensitive cameras is a minimally invasive approach to obtaining relative densities and estimating population trends in animals over time^1,2^. It enables researchers to study their subjects remotely by counting animals from the collected images^3^. However, due to their complexity, images are not readily analyzable in their raw form and relevant information must be visually extracted. Therefore, human labor is currently the primary means to recognize and count animals in images. This bottleneck impedes the progress of ecological studies that involve image processing. For example, in the Snapshot Serengeti camera-trap project, it took years for experts and citizen scientists to manually label millions of images^4^.

Deep-learning methods^5^ have revolutionized our ability to train digital computers to recognize all kinds of objects from imagery data including faces^6,7^ and wildlife species^4,8,9^ (see Appendix 1 for more background information). It may significantly increase the efficiency of associated ecological studies^4,10^. In our quest to demystify the method and increase the capabilities of machines to communicate with humans, it would be useful to have machines articulate the features they employ to identify objects^11,12^. This articulation would not only allow machines to converse more intelligently with humans, but may also allow machines to reveal weakness of the methods, dataset biases, and cues that humans are currently not using for object identification, which could then make humans more effective at such identification tasks. Before we can do this, however, we must identify the human-coherent, visual features used by machines to classify objects. To the best of our knowledge, none of the few existing studies that use deep learning for animal classification concentrate on this issue. As such, they lack the necessary transparency for effective implementation and reproducibility of deep learning methods in wildlife ecology and conservation biology.

To identify such features in the context of classification of wildlife from camera trap data, we trained a Convolutional Neural Network (CNN)^9,13^ using a deep learning algorithm (VGG-16, as described elsewhere^14^ and in Appendix 4) on a fully annotated dataset from Gorongosa National Park, Mozambique (Appendix 2) that has not previously been subjected to machine learning. For purposes of comparison, we repeated the training using a ResNet-50 CNN architecture, as discussed in Appendix 5. After training, we interrogated our network to better understand the features it used to make identifications by deconstructing the features on the following three aspects of our implementation: (1) localized visual feature, (2) common intraspecific visual features, and (3) interspecific visual similarities (Fig. 3 in Appendix 4).

We used Guided Grad-CAM (GG-CAM) methods–a combination of Guided Back-propagation (GBP)^15^ and gradient-weighted class activation mapping (Grad-CAM)^16^)–on the last convolutional layer of our trained network to extract localized visual features of single images. By inspecting the results, we could obtain indirect reasons of the CNN classifications. Next, we used the Mutual Information (MI) method^17,18^ to generalize within-species features as an extension of the localized visual feature of single images. These generalized features revealed inner biases of the dataset. Then, we used hierarchical clustering^19^ on the CNN feature vectors to further inspect the visual similarities between animals species learned by the CNN. We found that the relative visual similarities emerged during training process were similar to human knowledge. We also measured the relative familiarity of both “known” and “unknown” animals species to the CNN. The results implied that visual similarities could be used to identify visually distinct “unknown” animal species. Finally we conducted a relatively informal experiment that compared extracted features with visual descriptors used by human classifiers to identify species in our image sets of corresponding animal species. We found that, to some extent, the features used by the CNN to identify animals were similar to those used by the human. In the Discussion section, we provide a brief example of how interpretations of CNNs can help to understand the causes of misclassification and to make potential improvements of the method.

## Methods and Results

### Model training and localized feature visualization

**Figure 1:**
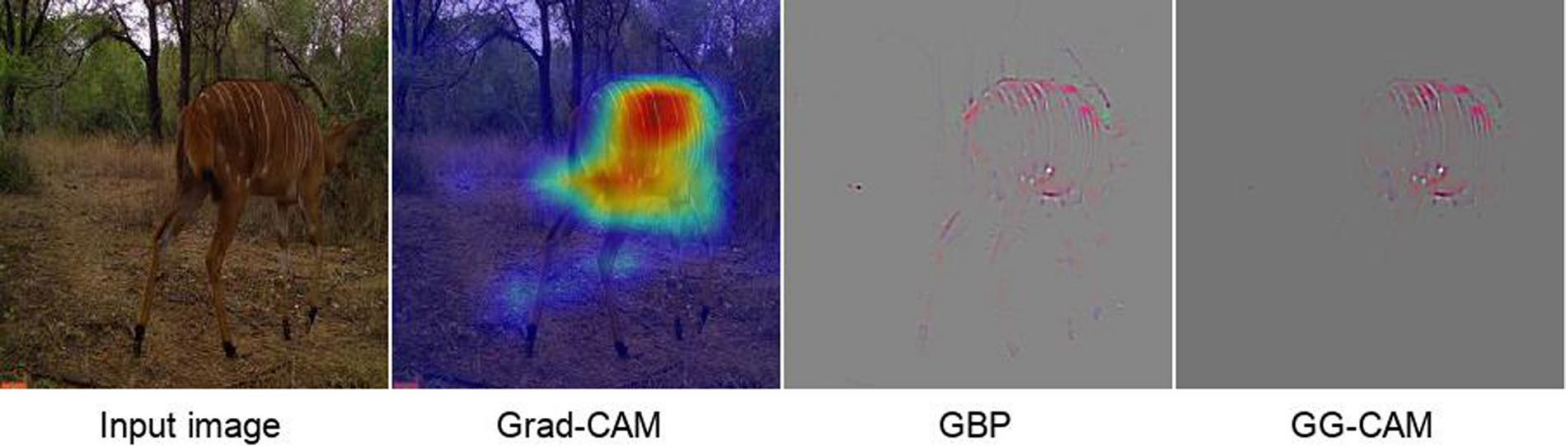
Comparison between Grad-CAM, GBP, and GG-CAM. Once trained, any image (leftmost panel) can be overlaid with its Grad-CAM heat map (left center panel) to identify the region of ‘most interest’ to the CNN (see Appendix 4). Similarly, the corresponding feature map (center right panel), produced using Guided Back-propagation (GBP), (which, as described in Appendix 4, identifies the most important visual features to our CNN) can be weighted by the Grad-CAM heat map to produce the guided Grad-CAM (GG-CAM) image seen in the rightmost panel. Note that in this Nyala image, GBP is less discriminative than GG-CAM: both highlight the stripes of the Nyala, whereas GPB includes non-species-discriminative tree branches and legs.

Before interpreting a CNN, we firstly trained a VGG-16^14^ and later a ResNet-50^20^ (Appendix 5) on a fully annotated dataset from Gorongosa National Park, Mozambique (Appendix 2). To increase the convergence fidelity of our learning algorithm in extracting species-specific visual features, we confined our training images to only the 20 most abundant species (ranging from 473 images of hartebeest to 28,008 images of baboons, Fig. 1 in Appendix 2). Adding some of the rarer species would have degraded the overall performance of the network because the network has fewer images to use in generalizing species-specific visual features^21^ (see Appendix 3 for more details).

**Figure 2:**
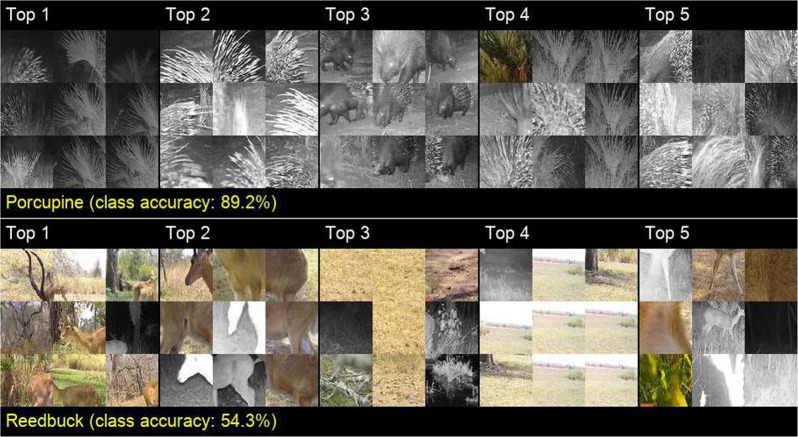
Image patches that respond most strongly to the five neurons with the highest MI scores of porcupine and reedbuck. The leftmost set of nine 60 × 60-pixel patches are extracted from nine camera-trap images that include a species of interest and have the highest responses to the corresponding neuron. In each of the nine cases, the extracted patches are centered around the “hottest” pixel (i.e., highest response) of the neuron (in the last convolutional layer of our CNN) that have the highest MI score (Appendix 4) for the said species class. The MI scores are calculated using 6000 randomly selected training images (300 images per class). The remaining four sets of nine patches are equivalently extracted for the neurons with the next four highest MI scores. These patches provide a sense of the within-species features to which the neuron in question responds. The higher the class accuracy, the more closely correlated these image patches are for the species of interest. For example, in the relatively accurately identified porcupine set (89.2% accuracy), the first neuron (Top 1, of the upper set) responds to palm plants that appear in most of the training images that also contain porcupines. The second neuron (Top 2) responds to the quills, while the third neuron (Top 3) responds most strongly to bodies with faces. On the other hand, in a much less accurately identified reedbuck set, the first neuron (Top 1, of the lower set) appears to respond to branch-like structures, including tree limbs and horns, but the patterns are less consistent than for the porcupine. Note that some sets of patches are primarily backgrounds (e.g., Top 1 upper set and Top 4 lower set), from which we can infer that our CNN learns to associate certain backgrounds with particular species. Such associations, however, only arise because particular cameras produce common backgrounds for all their images, thereby setting up a potential for a camera-background/species correlation that can well disappear if additional cameras are used to capture images. Similar sets of images are illustrated for other species in Fig. 9, Appendix 5.

Under this somewhat ad-hoc constraint on the number of species, after pruning out all images not containing the 20 most abundant species, we split the remaining 111,467 images at random into training (85% of images), validation (5% of images; for tuning hyperparameters listed in Table 1 in Appendix 3), and testing (10% of images; for evaluating accuracy) subsets. We used a deep learning algorithm (VGG-16)^14^ (see Appendix 3 for implementation details), which we then evaluated for accuracy once trained (Fig. 2 in Appendix 3; overall accuracy was 87.5%, and average accuracy across the 20 species was 83.0%, ranging from a high of 95.2% for civet to 54.3% for Reedbuck).

Then, we used GG-CAM methods, which combines the output from GBP^15^ and Grad-CAM^16^, on the last convolutional layer of our trained network, where feature localization occurs (see Appendix 4). We note that Grad-CAM captures the most discriminative image patch, GPB captures visual features both within and outside of the focal Grad-CAM patch, and GG-CAM captures the features most salient to the actual discrimination process (Fig. 1). When making correct classification, the CNN could extract species-specific features from the input images, such as the white spots and the white stripes of the Nyala in Fig. 1. We then inspected the GG-CAM images produced by our CNN relative to the original images in order to assess what sort of localized visual discriminative features were being extracted from the original images (Fig. 5); in this manner, we obtained information on the inner mechanism of deep learning classification^22,23^.

### Common within-species features

Next, we used the Mutual Information (MI) method^17,18^ to extend the features of single images to within-species features of each animal species. We calculated the MI scores for each of the neurons in the last convolutional layer of our CNN to indicate their importance to all images of one of the selected species (Appendix 4). In short, for each of these neurons, we obtained 20 species-specific MI scores from 6000 randomly selected training images (300 images of each species). For each species, we identified the five neurons in the last convolutional layer that produced the five highest scores. We then identified the top nine “hottest” 60 × 60 pixel patch (within-species features) to which each of these top five neurons responded in each image (e.g, Fig. 2 in Appendix 5). These features generalize across all images within the same species, as illustrated in Fig. 9 in Appendix 5. Most results are associated with distinguishable visual features of the animals, for example, black spots on civets, an elephant trunk, quills on porcupines, and white stripes on nyala.

However, visual similarities of animal species are not the only information our CNN uses to identify species. CNNs also use information such as the presence of trees in the background to identify species frequenting woodlands, especially when most of the images are from similar environments or the same camera-trap locals (e.g., image patches of the top1 neurons of wildebeest and porcupine in Fig. 9 in Appendix 5). These information reflects the inner bias of the dataset. For example, when most of the images of a class were taken from similar camera locals (i.e. backgrounds of the images could be similar), CNNs do not have to learn species-specific features during training, and the generality of the CNN can be largely degraded^24^. Reedbuck in Fig. 2 is another good example. Image patches of the Top 4 neuron are mostly the same. This is because that a large amount of the reedbuck images were taken by the same camera, which produced common backgrounds. Enhancing CNN’s ability to focus more on target objects/animals is a future direction to improve the generality of animal classification.

### Interspecific visual similarities

**Figure 3:**
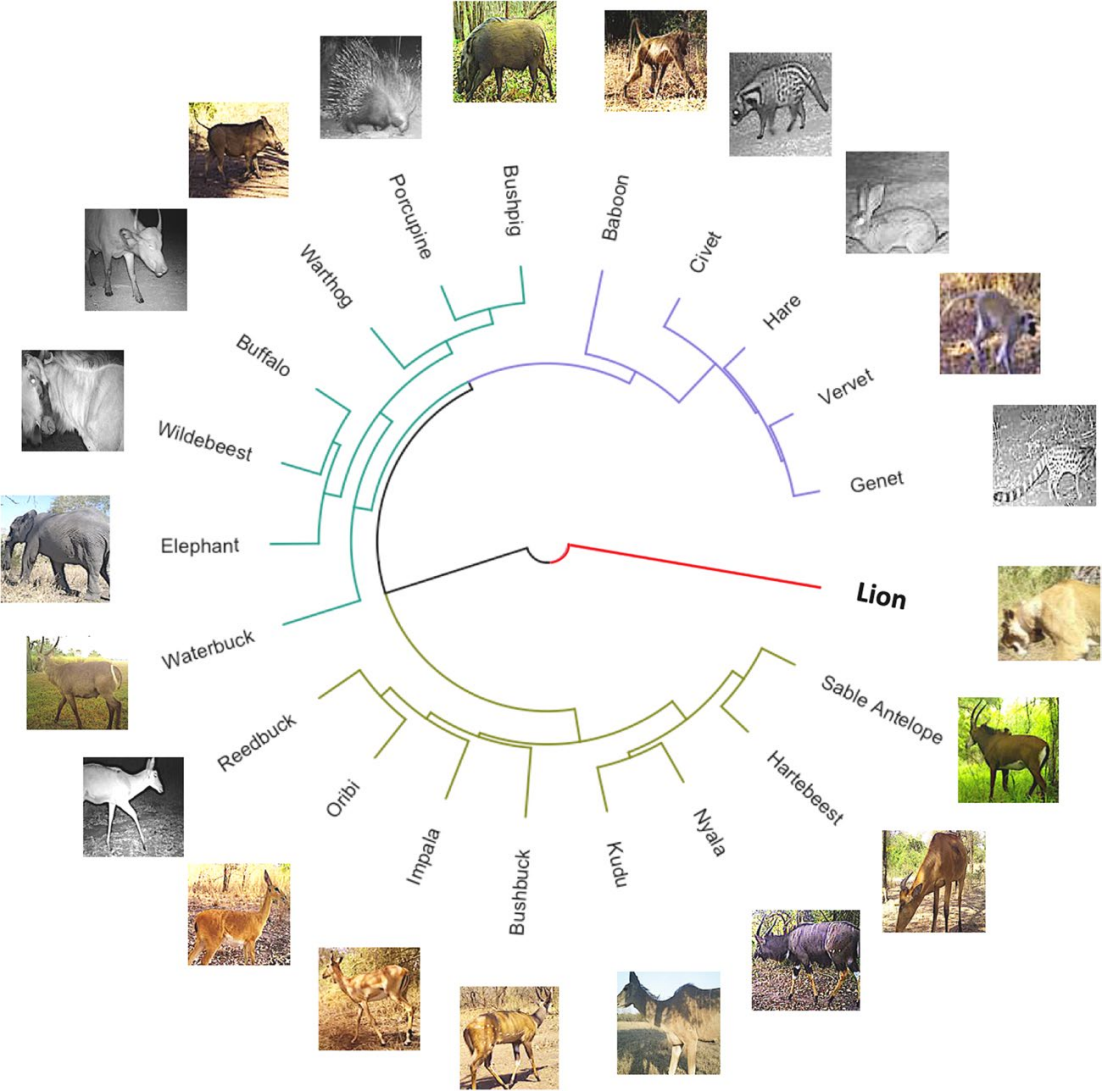
Visual similarity tree for our trained CNN. The similarity tree is based on hierarchical clustering of the response of the last fully-connected layer in our trained CNN to 6000 randomly selected training images of particular species (i.e., feature vectors of the images). The leaves represent feature vector centroids of 300 training images of each species, and their relative positions in the tree indicate the Euclidean distances between these centroids in the feature space. In the similarity tree, the more similar the response of this layer to two species, the more tightly coupled they are in the tree. Green, purple, and brown branches correspond to three primary clusters that appear to be a small to medium-sized antelope cluster, an animals-with-prominent-tail or big-ears cluster (though baboons seem to be an outlier in this group), and a relatively large body-to-appendages group (with waterbuck the outlier in this group). When the feature vectors of unkown animal species are placed in the tree (e.g., the red branch of lion), sometimes they can differ greatly from those of the known species.

We generated a visual similarity dendrogram for all species by applying hierarchical clustering^19^ to the CNN feature vectors of 6000 randomly selected training images, i.e., the outputs of the last fully-connected layer (which is of dimension 4096 in Euclidean space) of our trained CNN (see Appendix 4). This dendrogram (Fig. 3) is an abstract representation of how images of species are separated in the feature vector space. It also provides a means for quantifying how visually similar the 20 animal species are to our trained CNN. Similar animals are measurably closer together than those that are visually distinct (e.g., striped versus spotted; long-tailed versus no-tail), irrespective of their phylogenetic distance. Thus, though most of the antelopes are grouped together (from sable to reedbuck), the large bull-like herbivores (wildebeest and buffalo) and pig-like mammals (warthog, porcupine, and bushpig) are also grouped together even though they may belong to different families or orders (Fig. 3). A well-learned feature vector space can also help identify images that differ in some way from those on which the CNN has been trained^25,26^.

**Figure 4:**
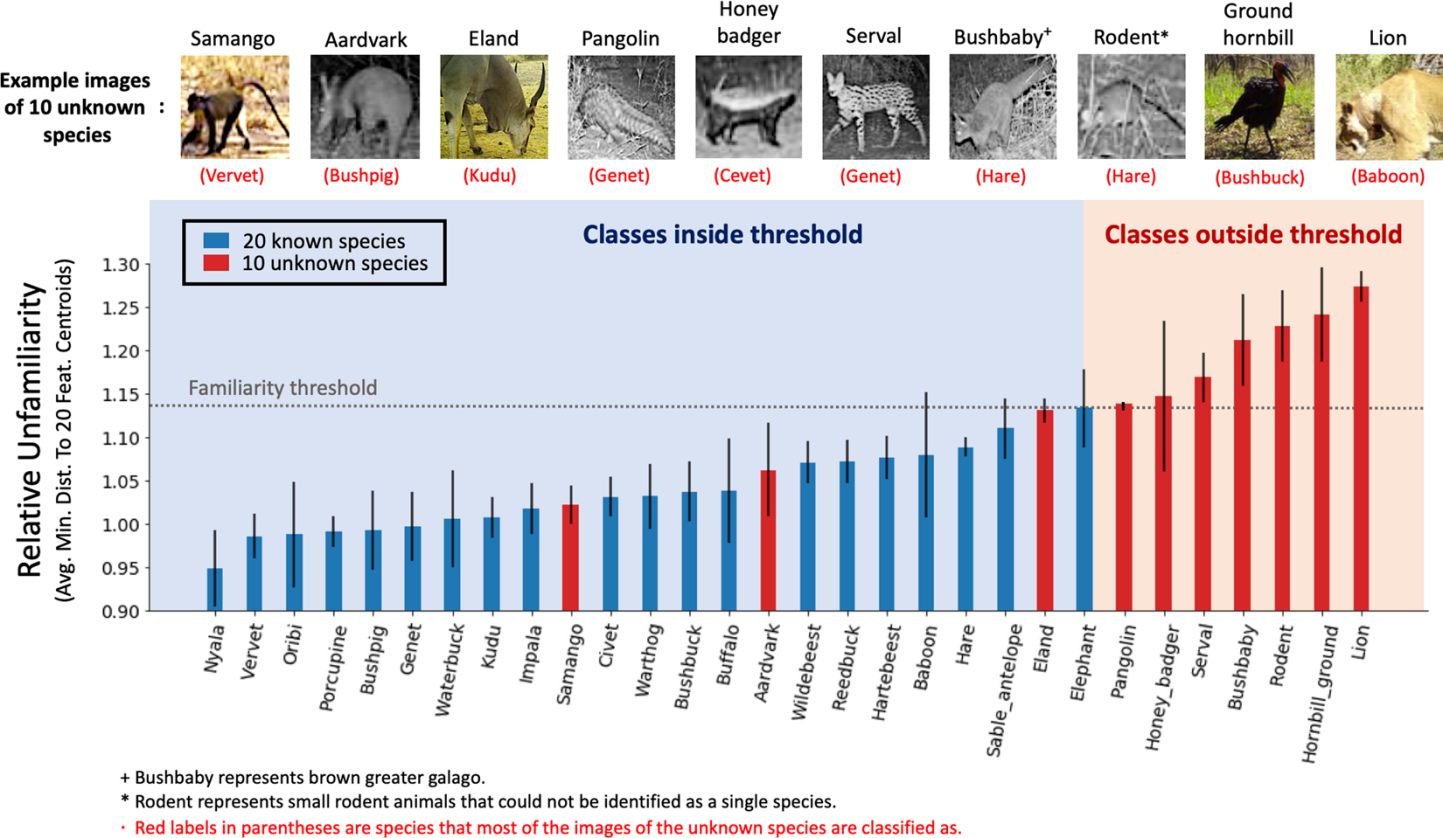
Relative unfamiliarity of 30 species (including 10 unknown species) to the CNN. Twenty species were used to train the CNN (known species–see Fig. 3) and then ten additional species (unknown species) were tested to see how their average feature vectors (averaged across 20 different exemplar photographs for each species–see text for details) fell within the feature vector space. Seven of the 10 unknown species had average feature vectors yielding a relative unfamiliarity value above our nominal“unfamiliarty threshold,” defined as the known species having the highest relative unfamiliarity value.

To measure the relative unfamiliarity of both known and unknown species to the CNN, we incorporated the 10 excluded rarer animal species into the testing data, and then implemented a 10-round random selection as follows. In each round, we randomly selected 20 testing images of the 30 animal species and then calculated the Euclidean distances of their feature vectors to the 20 feature-space centroids that were used to construct the dendrogram. The relative unfamiliarity of each class was calculated as the mean distance of the 20 testing images to their closest species feature-space centroids across the 10-round random selection (Fig. 4, also see Appendix 4). The intuition is that the more familiar the species were to the network the closer the average distances would be to one of the 20 feature-space centroids of training data. The known species had relative unfamiliarity values ranging from 0.95 to just over 1.1, with elephant being the largest at 1.14. We set this elephant value to be our nominal unfamiliarity threshold and found that seven of the 10 species fell above it (i.e., were less familiar to our trained CNN than any of the “known” species; viz., pangolin, honey badger, serval, bushbaby, rodent, ground hornbill, and lion) while three of the “unknown” species (viz., samango monkey, ardvark, and eland) appear to share features with the 20 known species (e.g., monkeyness: samango unknown and vervet known; antelopeness: eland unknown, hartebeest, wildebeest and sable known)^27^.

**Figure 5:**
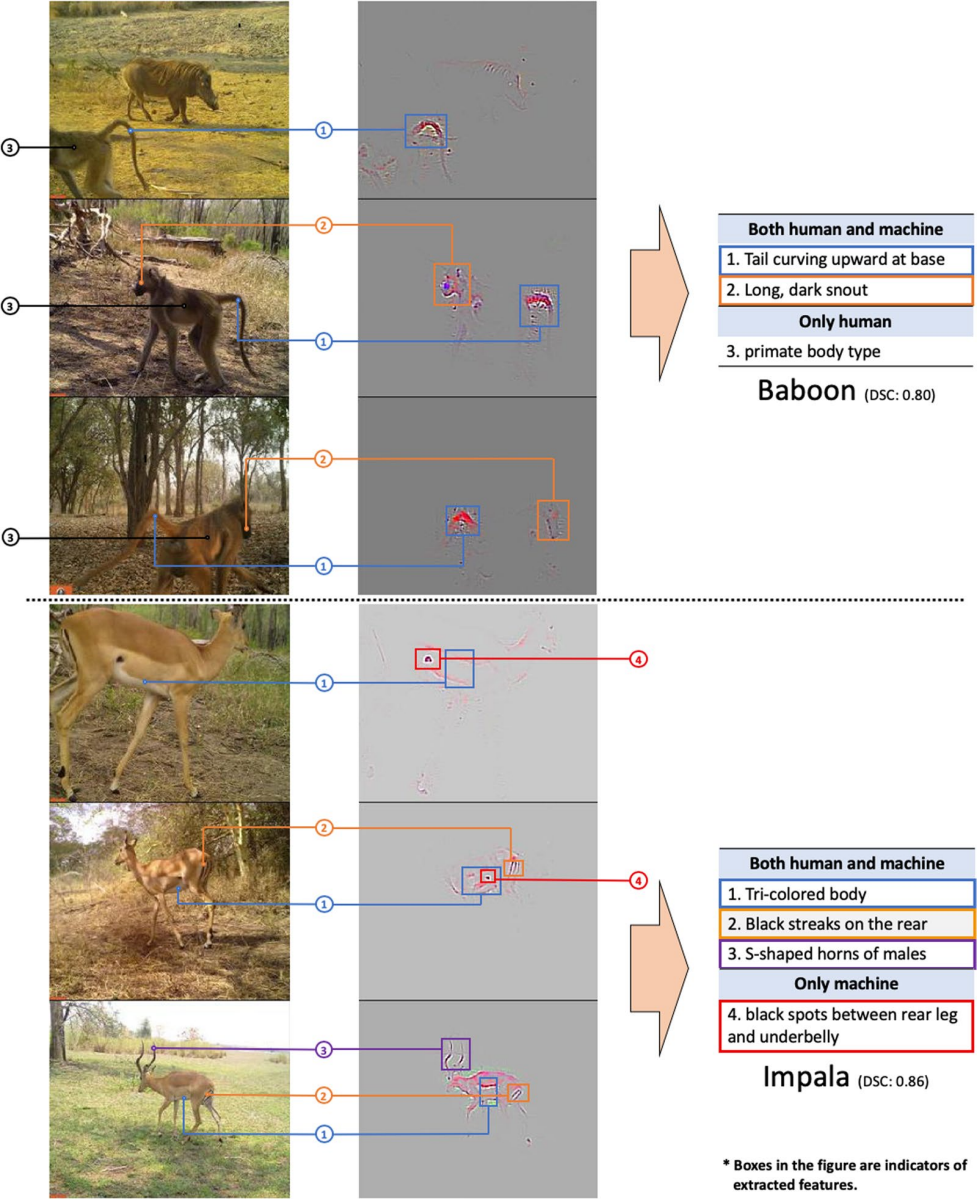
GG-CAM generated localized discriminative visual features of randomly selected images of baboon and impala. For classifying baboons, the CNN focuses on faces and tails. For impalas, the CNN uses the contrast between the white underbelly and dark back, black streaks on the rear, and black spots between the rear legs and underbelly. Most of the features extracted by the CNN have counterparts (similar focal visual components) in the human visual descriptors (indicated by the colors and agreed upon by at least 2 of 4 authors). The similarity is calculated as the DSC between extracted features and corresponding human descriptors (further detail in Fig. 8, Appendix 5).

### Features most salient to our team and trained observers

We conducted a relatively informal assessment of similarities and differences in the features extracted by GG-CAM to those most salient to some of our team, as described in Appendix 2 and Table 5 in Appendix 6. We did this by calculating the Dice similarity coefficient (DSC) for each species (see Appendix 4). The similarity mapping was agreed upon by at least two of four authors (ZM, KMG, ZL and MSN) who scored nine randomly selected images for each species. To some extent, the trained CNN uses features similar to those used by individuals trained to identify most of the animal species in our images (as presented in Fig. 8 in Appendix 5, where the mean DSC across species was 0.69 with standard deviation: 0.13). Figure 5-Baboon shows that our CNN uses faces and tails to identify Baboon images. Both of the two features have counterparts (similar focusing areas) in Table 5-Baboon in Appendix 6. In Fig. 5-Impala, besides the black streaks on the back ends, the line separating the colors of the upper body from the white underbelly and S-shaped horns, the CNN also appears to consider the black spots between the rear legs and bellies of impala as a discriminative feature. This feature, although not included in the most-used descriptors, is a good example of a discriminatory feature traditionally overlooked by us but now identified by our CNN as salient for use in future identifications. A more challenging example of Reedbuck can be found in Appendix 5.

### Comparison with ResNet-50

**Figure 6:**
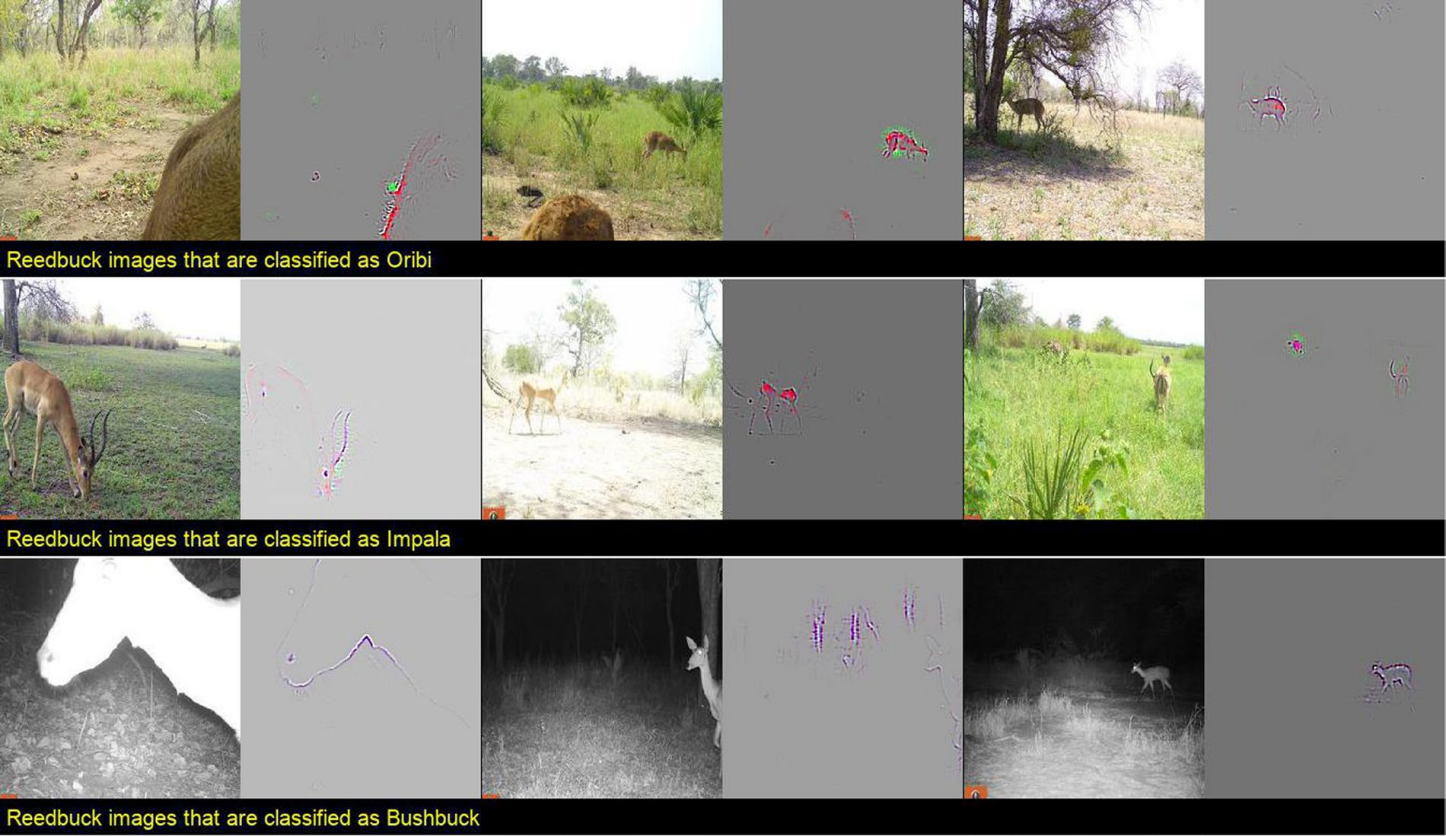
Examples of reedbuck images that are misclassified as oribi, impala, and bushbuck, with corresponding localized discriminative visual features. Although the CNN can locate animals in most images, it is hard for the machine to find distinct features from: (1) images with animals that are far away in the scene; (2) over-exposed images; (3) images that capture only parts of the animal; and (4) images with multiple animal species. In many of these cases, the other species are indeed present in the scenes, and are often in the foreground. This problem is an artifact of the current labeling process and remains to be resolved in the future. For example, the animal in the leftmost image on the second row that is classified as impala is an impala. The CNN correctly classifies this image based on the animal. However, this image was also labeled as reedbuck because the extremely small black spots far in the background are reedbuck. When two species appear in the same scene, the same image is saved twice in the dataset with different labels corresponding to different species in the scene. This labeling protocol can confuse the CNN and remains a problem that must to be resolved in the future.

To demonstrate the generalization of our observations, we also conducted comparison experiments using ResNet-50^20^, an algorithm with more layers, but fewer parameters than VGG-16 (see Appendix 5). In general, both of these two algorithms yielded similar results. For example, ResNet-50 extracted similar localized visual features to VGG-16 (e.g., tails and snouts of baboon and black spots and stripes of impala; Fig. 4, Appendix 5). It also appeared to extract similar within-species features of porcupines (e.g. quills, palm trees, and porcupine faces; Fig. 5-Porcupine, Appendix 5), although the hierarchical clustering results were somewhat different when comparing the two methods (Fig. 6, Appendix 5). As with VGG-16, ResNet-50 clustered most of the antelope animals together, but ResNet-50 had slightly better testing accuracy than VGG-16 (Table 4 in Appendix 5) and was more sensitive to edges when extracting localized visual features from individual images (Fig. 4, Appendix 5). When class accuracy is relatively low (e.g. reedbuck), ResNet-50 tended to extract more random within-species features (Fig. 5-Reedbuck, Appendix 5).

## Discussion

Understanding the mechanisms of deep learning classifications of camera-trap images can help ecologists determine the possible reasons for misclassification and develop intuitions about deep learning, which is necessary for method refinement and further implementation. For example, Fig. 2 in Appendix 3 indicates that reedbuck is the least accurately classified species by the CNN. The confusion matrix^28^ of testing results (Table 3, Appendix 3) reveals that many reedbuck images are classified as oribi (8%), impala (12%), and bushbuck (12%). Figure 3 shows that reedbuck is close to oribi, impala, and bushbuck in the feature vector space learned by the CNN, which partly explains misclassification. Further, by examining the localized visual features of the misclassified images, we can gain a clearer sense of the reasons for misclassification. Figure 6 depicts examples of misclassified reedbuck images. Although the CNN can locate the animals in most of the images, it is challenging for the CNN to classify the images correctly when the distinct features of the species are obscured or multiple species are in the same scenes.

## Conclusion

Deep learning has become a core component of data science and fields using big data. Ecology has been no exception, with its shift towards the machine learning methods in ecoinformatics^29,30^, including problems in conservation biology^31^, as well as the merging of data analytics with the scientific method^32^. This shift requires that new methods, including models from machine learning and artificial intelligence, are accessible and usable by ecologists^33^. Our paper provides practical steps in model interpretation to help ecologists take advantage of deep learning as a cutting-edge approach for future research and for overcoming major methodological roadblocks. The interpretations described in this paper are steps toward a more informed use of deep learning methods. Future research involving the training of CNNs to identify individuals in ecological studies, whether for purposes of species classification, conservation biology, sustainability management, or identification of specific individuals in their own right^34,35^ (e.g., in behavioral studies) can follow the methods presented here to identify the sets of features being used to classify individuals. This information may then be used in creative ways yet to be imagined to improve CNN training and, hence, raise the level of performance of CNNs as an aid to analyzing ecological data.

## Supplementary information

Supplementary material: Insights and approaches using deep learning to classify wildlife
