# Supplementary material for: Insights and approaches using deep learning to classify wildlife
Source: Sci Rep. 2019 May 31;9:8137. doi: 10.1038/s41598-019-44565-w (PMC6544615; doi:10.1038/s41598-019-44565-w)
Supplement: Supplementary file 1 — Supplementary material: Insights and approaches using deep learning to classify wildlife [file 41598_2019_44565_MOESM1_ESM.pdf]

Supplementary material: Insights and approaches using deep learning to classify wildlife

Zhongqi Miao[1,2*], Kaitlyn M Gaynor[1], Jiayun Wang[2,3], Ziwei Liu[2], Oliver Muellerklein[1], Mohammad Sadegh Norouzzadeh[4], Alex McInturff[1], Rauri C K Bowie[5], Ran Nathan[6], Stella X Yu[2,3], and Wayne M Getz[1,7*]

[1]Dept. Env. Sci., Pol. & Manag., UC Berkeley, CA, United States
[2]International Comp. Sci. Inst., UC Berkeley, 1947 Center St, Berkeley, CA, United States
[3]Vision Sci. Grad. Group, UC Berkeley, CA, United States
[4]Dept. Comp. Sci., U. Wyoming, WY, United States
[5]Dept. Integr. Biol. & Museum of Vertebrate Zoology, UC Berkeley, CA, United States
[6]Dept. EEB, Alexander Silberman Inst. Life Sci., Hebrew U. Jerusalem, Givat Ram, Israel
[7]Sch. Math. Sci., Univ. KwaZulu-Natal, South Africa
[*]zhongqi.miao@berkeley.edu
[*]wgetz@berkeley.edu

Appendix 1: Background

Camera trap studies

Camera traps have become an increasingly popular tool for the remote monitoring of wildlife populations[1]. They are a low-cost method for gathering data on an entire wildlife community, and are especially useful for studying rare, cryptic, or nocturnal species, including many species of conservation concern[2]. Long-term camera trap datasets can be useful for monitoring trends in populations over time, and novel analyses enable the estimation of relative densities and abundances across time and space[3]. Camera traps set across spatial gradients of environmental heterogeneity can also be used to understand environmental and anthropogenic drivers of wildlife distributions. Analyses can range from simple statistical tests (e.g., Analysis of Variance of relative activity across habitat types) to complex models (e.g., Bayesian hierarchical occupancy models that account for imperfect detection and incorporate multiple predictors of occupancy and detection[4,5];). Finally, images or videos from camera traps provide insight into animal behavior, including movement and migration patterns, foraging and anti-predator vigilance, or reactions to experimental stimuli[6]. However, in all of these cases, camera trap studies are limited by the inefficiencies of data processing and the manual classification of species and behaviors in images. Deep learning with CNNs has proven to drastically improve the efficiency of relevant studies. In this paper we demonstrate the mechanisms of deep learning in detail.

Deep learning in camera-trap classification

Deep learning is a subdomain of machine learning that uses algorithms inspired by biological neural networks[7]. It has gained much attention among ecologists in recent years[8], with animal species identification from camera trap images using CNNs being one of the most popular applications[9–14]. Chen et. al.[9] made the first attempt to automatically classify camera trap images with deep learning methods. They achieved only 38% classification accuracy on their 20,000-image dataset, and suggested that, with enough training data, deep learning can surpass other existing methods. Gomez et. al.[11] harnessed deep learning with transfer learning, a method of fine-tuning, to identify animal species in the Snapshot Serengeti dataset[15,16] and achieved over 80% classification accuracy using large amounts of data. Further, Norouzzadeh et. al.[10] trained multiple CNN architectures on the same dataset as Gomez et. al. and achieved a classification accuracy in excess of 95%, the current state-of-the-art performance for deep learning models in camera-trap studies. However, to our knowledge, there are no studies specifically explaining the mechanisms of deep learning that facilitate classification of animals with such a high degree of accuracy. In addition, the only big camera-trap dataset to which this method has been applied is the Snapshot Serengeti dataset. In this paper, we implement deep learning on a dataset that has not been studied previously and illustrate three approaches to interpretation to reveal the mechanisms of CNNs qualitatively.

Basic mechanisms of CNNs

Convolutional neural networks (CNNs) are one of the most frequently used deep networks in computer vision From AlexNet[17] to VGG[18] and ResNet[19], the capacity of modern CNN architectures has advanced rapidly, resulting in high recognition accuracies that make abundant real-world applications possible. Modern CNN architectures typically have three types of layers – convolutional layers, pooling layers and fully-connected layers – which gradually transform an input image into a predicted category label. For instance, the VGG-16 network (architecture used in this paper) has 13 convolutional layers, 5 pooling layers, and 3 fully-connected layers; it takes a 227×227 image as input and predicts a 1-of-1000 category label as output. Convolutional layers in CNNs consist of local filters or neurons and are designed to capture spatially-distributed local traits such as edges, parts and textures[20]. Pooling layers account for the larger receptive field of the deeper convolutional layers, i.e. the subsequent convolutional layers assemble the previously learned local traits into more globally-perceived shapes and configurations[21]. Fully-connected layers abstract all of the local and global traits into high-level semantic concepts like categories and attributes[22]. All the parameters in the CNNs are learned by minimizing the errors between prediction and ground-truthed data through a layer-by-layer updating process called back-propagation. In this work, we interpret the inner representations of CNNs qualitatively and quantitatively by examining the relationship between neurons and ecological data.

Interpretable deep learning

Though deep learning achieves impressive accuracy on many visual recognition tasks, its "black-box" mechanism makes it hard for users to understand the underlying inference process. To alleviate these drawbacks, researchers develop various methods towards interpretable deep learning by decomposing and organizing the internal learned features. Representative works include GG-CAM[23] and network dissection[24], which localize and extract meaningful parts and regions that are coherent to human perception and reasoning[25]. The interpretable deep learning techniques have been successfully applied to face analysis[22], scene understanding[21], chest radiograph diagnosis[26] and plant species identification[27,28]. In this work, we leverage the recent advances in interpretable deep learning to shed light on the deep learning based wildlife classification, which provide useful practices and new insights for future deployment in ecology-related fields.

Appendix 2: Data

Data collection

The camera-trap data comes from a long-term research program in Gorongosa National Park, Mozambique (18.8154°S, 34.4963°E). The dataset used in this analysis was collected from June to November of 2016. The goal of this program is to examine the spatial distribution of large mammal species in the park and to monitor the restoration of the park's wildlife following decades of civil war. The 3,700 km^2 park encompasses a range of habitats, including a mix of grassland, open woodland, and closed forest. KMG placed 60 motion-activated Bushnell TrophyCam and Essential E2 cameras in a 300 km^2 area in the southern area of the park. Each camera was mounted on a tree within 100 meters of the center of a 5 km^2 hexagonal grid cell, facing an animal trail or open area with signs of animal activity. To minimize false triggers, cameras were set in shaded, south-facing sites that were clear of tall grass. Cameras were set to take 2 photographs per detection with an interval of 30 seconds between photograph bursts. *All the photographs of animals used in this paper are from this dataset.*

Trained researcher classification

The species in all images were classified and manually annotated independently by two different researchers trained on a list of example images and corresponding visual descriptors of each species; this list was created by KMG before the manual annotation and was iteratively updated as the annotation progressed. All classifications were confirmed by KMG prior to this project.

We conducted a survey to determine the features that were regularly used by humans to identify each of the 20 species in this study. For each species, respondents were asked to select features that they regularly look for and/or use as clear diagnostic features that identify the species, and could select as many descriptors as they wanted. We provided respondents with KMG's list of all visual descriptors used in training materials, and included an option of adding additional descriptors not mentioned. The survey had 13 respondents, all of whom have extensive experience classifying camera trap images from Gorongosa National Park, including those used in this study. KMG selected the participants, and they included 5 undergraduate research apprentices, 3 other researchers affiliated with Gorongosa National Park, and 3 trained citizen scientists. KMG and ZM also completed the survey. We considered a feature to be regularly used by humans if at least 5 of the 13 respondents selected it (Table 5).

Data description

The dataset contains a total of 30 animal species. In this paper, we use data from the 20 most commonly photographed mammal species during data collection for higher training performance and more accurate feature extraction (Figure 1) and omit rare species with less than 350 images, as well as images that are annotated as empty (Figure 1) during training and performance testing. The 20 species include: African buffalo (*Syncerus caffer*); African elephant (*Loxodonta africana*); African savanna hare (*Lepus microtis*); baboon (*Papio cynocephalus*); blue wildebeest (*Connochaetes taurinus*); bushpig (*Potamochoerus larvatus*); Cape bushbuck (*Tragelaphus sylvaticus*); civet (*Civettictis civetta*); southern reedbuck (*Redunca arundinum*); crested porcupine (*Hystrix cristata*); greater kudu (*Tragelaphus strepciseros*); impala (*Aepyceros melampus*); large-spotted genet (*Genetta tigrina*); Lichtenstein's hartebeest (*Alcelaphus buselaphus*); nyala (*Tragelaphus angasii*); oribi (*Ourebia ourebi*); sable antelope (*Hippotragus niger*); vervet monkey (*Chlorocebus pygerythrus*); warthog (*Phacochoerus africanus*); and waterbuck (*Kobus ellipsiprymnus*). Besides the labels, each image has information on camera shooting times and animal habitat. There are seven types of habitat in the dataset: sparse woodland; sparse to open woodland; open woodland; open to closed woodland; closed woodland; closed woodland to forest; and forest.

When inspecting the relative familiarity of known and unknown animal species, we incorporated the 10 excluded species into the testing dataset. These 10 species are: aardvark (*Orycteropus afer*); bushbaby / brown greater galago (*Otolemur crassicaudatus*); eland (*Taurotragus oryx*); honey badger (*Mellivora capensis*); lion (*Panthera leo*); samango (*Cercopithecus albogularis*); serval (*Leptailurus serval*); southern ground hornbill (*Bucorvus leadbeateri*); Temminck's ground pangolin (*Smutsia temminckii*); and rodent (multiple rodent species).

The total number of images of the 20 most common animal species used in this project was 111,467. There are 6596 images (around 6% of the dataset) have multiple animals in the the same scene. We do not have specific preprocessing to the multi-label images. The reason is that we want the whole training process to be as realistic as possible, where misclassifications caused by current data storage protocols of multi-label data can happen.

Data preprocessing

We first grouped the images by camera shooting events. At each shooting event, when the motion sensors detect motion, the cameras captured two sequential images within one second. Image pairs of the same shooting events often are similar in appearance, and the training performance of the model can be overestimated if images from the same image pair are separated into training and test sets. Thus, we maintained the image pairs in the analysis in order to prevent a negative bias of the CNN

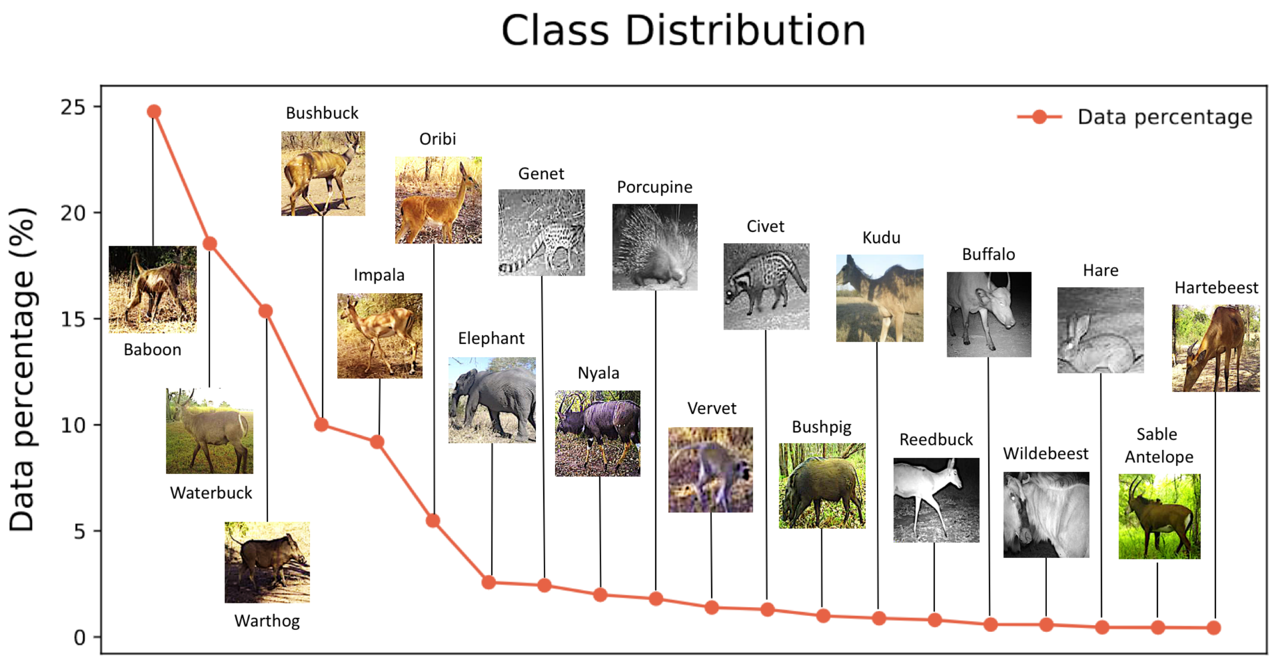

Figure 1. Distribution of 20 different animal species in the 111,467 images used to train, validate and test (85%, 5% and 10% respective split) our CCN. More than 60% of the images include the first three species. The overall accuracy of CNN was 87.5% and average accuracy across the 20 samples was of 83.0% (range was Civet 95.2% - Reedbuck 54.3%, as detailed in Fig 2)

learning process. We then randomly split the image groups into training, validation and testing sets with 85%, 5% and 10% of the datasets.

Appendix 3: Training details

Model implementation

We trained a VGG-16[18] CNN architecture to classify camera-trap images with class-aware sampling[29]. The output of the CNN classifier is a 20-dimensional vector, with each dimension representing the classification probability for an animal species (classification score). The use of class-aware sampling helps to improve classification accuracy for unbalanced datasets.

We made use of PyTorch[30], a deep learning framework, to implement and train the CNN. The weights were initialized from an ImageNet[31] pretrained model. The initial learning rate was 0.01, which decreased every 15 epochs. The best model was obtained at epoch 40 where the classification accuracy on the validation dataset was the highest. The loss function used to train the CNN was Softmax cross-entropy loss. All the input images for training were firstly downsized to 256×256, then were randomly cropped to 224×224 with a random horizontal flip at rate 0.5. Values of the hyperparameters used for training are listed in Table 1.

Table 1. Hyperparameters

Parameters	Values
Input image size:	256×256
Random crop size:	224×224
Random horizontal flip rate:	0.5
Batch size:	256
Training epoch:	40
Initial learning rate:	0.01
Momentum:	0.9
Learning rate reduce at:	every 15 epochs
Learning rate reduce by:	0.1
Regularization:	None

Assessing accuracy

The classification accuracies of the model are measured by both micro-averaged accuracy (over-all accuracy) and macro-averaged accuracy (averaged accuracy per class) in the testing set (Table 2). Figure 2 depicts classification accuracy by animal species.

Table 2. Testing accuracy

Metric	Accuracy (20 classes)	Accuracy (30 classes)
Overall micro accuracy:	87.5 %	73.7 %
Overall macro accuracy:	83.0 %	52.4 %

Experiment using 30 classes

For comparison, we also trained a VGG-16 model using the whole dataset (including the excluded 10 rarer animal species). Table 2 shows that with the 10 rarer animal species, the testing micro accuracy degraded by 14% and the testing macro accuracy degraded by 30%. This was because the model was unable to generalize discriminative features from limited amount of training data. This pattern can also be observed in the 20-class experiment (Table 3), where dominant classes have high recall but low precision.

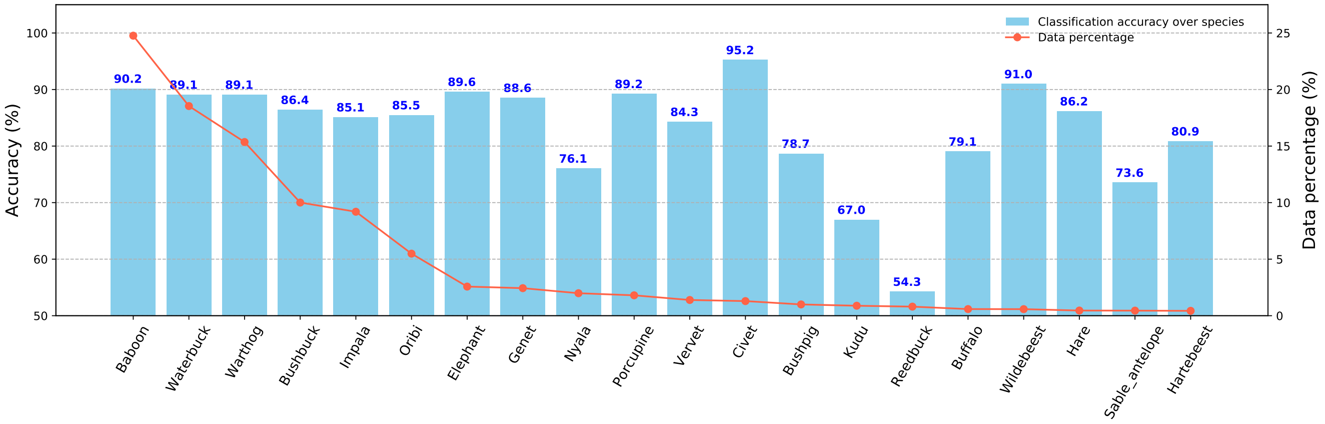

Figure 2. Model performance. Animal classification accuracy and data distribution per species. Reedbuck and Kudu are the two species for which the results were least accurate.

Confusion matrix

From Table 3 we can see that classes with dominant amount of data (e.g. Baboon, Waterbuck, and Warthog) show high recall but low precision. This is because the model cannot generalize discriminative features from classes with limited amount of training data, and thus data from these classes are more likely to be classified as dominant classes and cause higher false positive rates. This pattern has been discussed in detail by imbalanced classification and long-tail distribution studies such as[32].

Table 3. Confusion matrix in percentage (%), where the rows represent actual classes and columns represent predicted classes[33]. The numeric column headings represent: 0: baboon, 1: buffalo; 2: bushbuck; 3: bushpig; 4: civet; 5: elephant; 6: genet; 7: hare; 8: hartebeest; 9: impala; 10: kudu; 11: nyala; 12: oribi; 13: porcupine; 14: reedbuck; 15: sable Antelope; 16: vervet Monkey; 17: warthog; 18: waterbuck; 19: wildebeest.

	0	1	2	3	4	5	6	7	8	9	10	11	12	13	14	15	16	17	18	19
0	90.2	0.2	0.5	0.1	0.0	0.0	0.0	0.0	0.0	1.5	0.0	0.8	0.6	0.0	0.1	0.0	0.4	2.9	2.3	0.0
1	4.7	79.1	2.0	0.0	0.0	4.7	0.0	0.0	0.7	0.0	0.0	0.0	0.0	0.7	0.0	2.7	0.0	0.7	4.1	0.7
2	2.6	0.0	86.5	0.2	0.3	0.4	0.5	0.6	0.0	1.6	0.3	0.8	1.5	0.5	0.4	0.0	0.0	1.2	2.5	0.0
3	2.9	0.0	5.3	78.7	0.0	0.0	0.8	0.0	0.0	0.8	0.4	0.0	2.0	1.6	0.0	0.0	0.0	4.1	2.5	0.4
4	0.7	0.0	1.1	1.1	95.2	0.0	0.4	0.0	0.0	0.0	0.0	0.0	0.0	0.0	0.0	0.0	0.0	1.5	0.0	0.0
5	2.6	0.0	1.3	0.2	0.0	89.6	0.2	0.3	0.0	0.2	0.0	0.0	0.0	0.3	0.0	0.3	0.0	3.0	1.8	0.2
6	0.0	0.0	2.9	0.6	1.0	0.0	88.6	1.1	0.0	0.4	0.0	0.2	1.3	1.5	0.4	0.2	0.0	1.0	1.0	0.0
7	0.0	0.0	5.7	0.0	0.0	0.0	2.3	86.2	0.0	0.0	0.0	0.0	2.3	1.1	0.0	0.0	0.0	0.0	2.3	0.0
8	1.1	1.1	1.1	0.0	0.0	0.0	0.0	0.0	80.9	4.5	1.1	2.2	0.0	0.0	1.1	0.0	0.0	0.0	6.7	0.0
9	3.7	0.0	1.4	0.2	0.0	0.1	0.0	0.0	0.0	85.1	0.1	0.6	2.1	0.0	0.5	0.0	0.7	1.2	4.1	0.1
10	9.2	0.0	1.1	1.1	0.0	0.0	0.0	0.0	0.0	3.2	67.0	3.8	1.1	0.0	0.0	0.0	0.0	3.8	9.2	0.5
11	4.1	0.0	6.9	0.0	0.0	0.5	0.0	0.0	0.0	3.9	1.4	76.0	0.0	0.2	0.0	0.5	0.2	0.2	5.8	0.2
12	2.4	0.0	3.4	0.0	0.0	0.0	0.3	0.2	0.2	2.6	0.3	0.0	85.5	0.2	0.5	0.0	0.0	1.7	2.8	0.0
13	0.8	0.0	3.5	1.1	0.0	0.3	1.9	0.5	0.0	0.8	0.0	0.0	0.0	89.3	0.0	0.0	0.0	0.5	1.3	0.0
14	1.1	0.0	11.4	0.0	0.0	0.0	1.7	0.0	0.0	11.4	0.6	0.6	8.0	0.0	54.3	0.0	0.0	0.6	10.3	0.0
15	1.7	3.3	3.3	0.8	0.0	0.8	0.0	0.0	1.7	1.7	0.8	0.8	0.0	0.0	0.0	73.6	0.0	1.7	9.9	0.0
16	8.0	0.0	0.6	0.0	0.0	0.0	0.0	0.0	0.0	1.5	0.0	0.9	0.3	0.0	0.0	0.0	84.3	1.2	3.1	0.0
17	4.8	0.1	0.9	0.2	0.1	0.3	0.0	0.0	0.0	0.9	0.0	0.2	0.8	0.0	0.1	0.0	0.4	89.1	1.9	0.1
18	2.3	0.1	1.2	0.0	0.0	0.2	0.0	0.1	0.0	2.0	0.1	0.1	0.5	0.0	0.2	0.1	0.4	3.2	89.1	0.1
19	0.0	0.0	0.0	0.0	0.0	0.0	0.0	0.0	2.7	0.0	0.0	0.0	0.0	0.0	0.0	0.9	0.0	0.9	4.5	91.0

Appendix 4: Interpretation methods details

Outline of our general approach

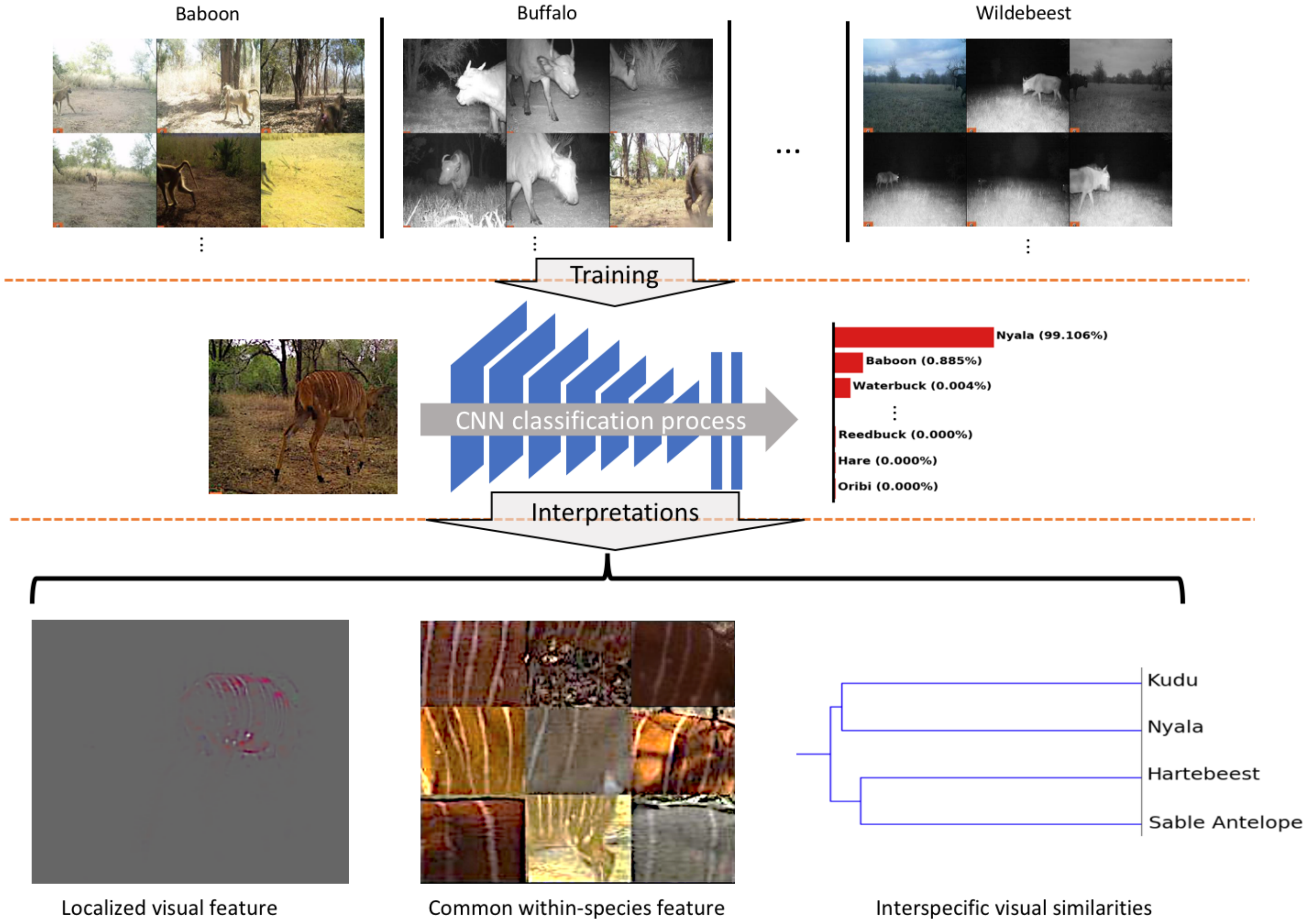

Figure 3. Overview of training and interpretations. We use the VGG-16 algorithm to train a convolutional neural network (CNN) on a camera-trap dataset collected from Gorongosa National Park, Mozambique. When a trained CNN is fed with images, it classifies the images with an estimated classification probability. To interpret the mechanisms behind this process, we use Guided Grad-CAM (GG-CAM) to find the localized visual features extracted by the CNN from the images. Next, we use Mutual Information (MI) on the neurons of the last convolutional layer to generate common within-species features of each species. Finally, we use hierarchical clustering on the feature vectors to study the relative interspecific visual similarities of each species in the dataset.

Guided Grad-CAM (GG-CAM)

GG-CAM is a method that combines the output of Grad-CAM and GBP[23]:

Grad-CAM generates coarse, discriminative regions according to animal species. It is calculated as the rectified linear units (i.e., that is the function $\max\{0,x\}$) of the weighted sum of the response maps from the last convolutional layer (Eq. 1). The weighted sum is based on the importance value α_k (importance value of the k_{th} neuron) of each neuron (neuron importance) in that layer of the response map, A^k, where its ij^{th} element is A^k_{ij} for a total number of elements Z. If y is the prediction score of animal A before the Softmax layer, then Grad-CAM is computed using the following equations:

$$\text{Grad-CAM} = \max\left\{0, \sum_k \alpha_k A^k\right\} \quad (1)$$

$$\alpha_k = \frac{1}{Z}\sum_i \sum_j \frac{\partial y}{\partial A^k_{ij}} \quad (2)$$

GBP is a method that captures non class-discriminative details of visual components that are important to the network overall. It is calculated as the gradient of the output response map of the last convolutional layer with respect to the input

image, with only positive gradients and positive response elements (Eq. 3). If R^l is the GBP product of the lth layer then it is calculated in terms of the response maps f^l of the lth layer, and the response maps f^{out} of the last convolutional layer. Specifically, defining $f^{l'} = \max\{0, f^l\}$, the equation is:

$$R^l = f^{l'} \times \max\left\{0, \frac{\partial f^{\text{out}}}{\partial f^{l'}}\right\} \times \frac{\partial f^{\text{out}}}{\partial f^{l'}} \tag{3}$$

After training the model, we can fix the model weights and use Eq. 1 to calculate the Grad-CAM of the input. We can also use (Eq. 3) to generate the GBP results of the inpurt with the trained model. Once Grad-CAM and GBP are both generated, we can calculate the Hadamard product (a.k.a. element wise multiplication) of Grad-CAM and GBP. GG-CAM is the normalized output of the Hadmard product (Figure 1, main text).

Mutual information

Next, we demonstrate another approach to inspect within-species animal discriminative features based on common neuron importance. Each neuron in the network has a response to certain parts of the input images. Classification of images is based on a combination of the neuron responses. In addition, per species, certain neurons are more important than others for classification. We assume that the responses from these neurons can be regarded as common within-species features.

We use Mutual Information (MI)[34], a method commonly used to find information shared between variables[35,36], on the neuron importance (Eq. 2) (normalized from 0 to 1) from the last convolutional layer across the data (Eq. 4). We calculated $I(U < C)$, the MI for neuron U and animal species C, as follows. Suppose N_{11} and N_{01} are the number of images of C, where U has neuron importance > 0.5 and ≤ 0.5 respectively. Further suppose N_{10} and N_{00} are the number of images that are not C, where U has neuron importance > 0.5 and ≤ 0.5 respectively. Defining $N_{1\cdot} = N_{10} + N_{11}$, $N_{\cdot 1} = N_{11} + N_{01}$, $N_{0\cdot} = N_{00} + N_{01}$, $N_{\cdot 0} = N_{00} + N_{10}$, and $N = N_{00} + N_{01} + N_{10} + N_{11}$ it then follows that

$$\begin{aligned} I(U,C) = & \frac{N_{11}}{N} \log_2 \frac{NN_{11}}{N_{1\cdot}N_{\cdot 1}} + \frac{N_{01}}{N} \log_2 \frac{NN_{01}}{N_{0\cdot}N_{\cdot 1}} \\ & + \frac{N_{10}}{N} \log_2 \frac{NN_{10}}{N_{1\cdot}N_{\cdot 0}} + \frac{N_{00}}{N} \log_2 \frac{NN_{00}}{N_{0\cdot}N_{\cdot 0}} \end{aligned} \tag{4}$$

We calculated the class wise MI scores using 6000 randomly selected images (300 images per class). Each neuron has 20 different MI scores with respect to each class. After the calculation, we selected nine images of each class that had the highest responses to each neuron. The results are illustrated in Figure 9 for image patches with the highest responses to the neurons with the top 1 to top 5 mutual information scores of each species.

Interspecific visual similarities and species familiarity

To inspect visual similarities between animal species, we generated a visual tree of all species by implementing hierarchical clustering on the feature vectors before the classifier layer (i.e. the output of the last fully-connected layer before the classifier layer). Firstly, we extracted the feature vectors of 6000 randomly selected training images and applied Principal Component Analysis (PCA) to compress the 4096-dimension feature vectors to 128 dimensions for computational simplicity. We then computed the average interspecific Euclidean distances between every pair of the 20 species. Finally, we processed the interspecific distances using a hierarchical clustering method with the Ward variance minimization algorithm[37]. The leaves in the dendrogram can be regarded as the feature vector centroids of the 20 classes (Figure 3, main text).

To calculate the relative unfamiliarity of 30 animals species, we first incorporated images of the 10 excluded species into the testing dataset, and performed a 10-round random selection. In each round, we randomly selected 20 images. This was because we only had 28 images for the rarest species (pangolin), and we wanted to keep the testing data balanced. We calculated the Euclidean distances of the feature vectors of these images to the 20 feature-space centroids that constructed the dendrogram. And, the relative unfamiliarity was calculated as the mean distance of these feature vectors to their "closest" centroids across the 10-round random selection.

Feature similarity calculation

To calculate the similarity between extracted features and human descriptors, we firstly did the feature mapping, which was agreed upon by up to four authors (ZM, KMG, ZL and MSN). Then we used Dice similarity coefficient (DSC) to calculate the similarity between two sets of features. Suppose M_C and H_C are machine extracted feature set and human descriptor set of animal C, the DSC are calculated as:

$$\text{DSC} = \frac{2 \cdot |M_C \cap H_C|}{|M_C| + |H_C|} \tag{5}$$

$|\cdot|$ is the cardinality of the sets.

Appendix 5: Additional results

Comparisons with ResNet-50

Here we present the results using ResNet-50[38], another widely used CNN architecture in computer vision with much deeper layers but fewer parameter numbers compared to VGG-16, to demonstrate the generalization of our observations in the main paper. Firstly, the classification accuracies are reported in Table 4. The testing performance of ResNet-50 was slightly better than VGG-16, with a 0.5% improvement on the micro accuracy and a 0.2% improvement on the macro accuracy.

Table 4. Testing accuracy of ResNet-50 and VGG-16

Metric	Accuracy (ResNet-50)	Accuracy (VGG-16)
Overall micro accuracy:	88.1 %	87.5 %
Overall macro accuracy:	83.2 %	83.0 %

Next, we performed the GG-CAM analysis using the trained ResNet-50 model on the same input images in Figure 4, main text. From Figure 4 we observe that both ResNet-50 and VGG-16 extracted similar visual features. ResNet-50, as mention in the original paper[38], was more sensitive to edges compared to VGG-16. And for the baboon images, it was less sensitive to the warm colors.

Then, we used the same set of images for the VGG-16 MI experiments to generate the MI results for ResNet-50. By comparing the image patches (Figure 5), we observe that, although different by appearance, ResNet-50 can also identify quills (Top 3 and Top 5) and heads (Top 1). For reedbuck images, the extracted features are relatively random, and the class accuracy was 1.2% lower.

Finally, we used the same set of images used for interspecific similarity experiments to generate hierarchical clustering results for ResNet-50. ResNet-50, however, appears to be not as good as VGG-16 in forming an “antelope group” (red branches) of species since it includes both baboon and warthog in this grouping.

From these three comparative scenarios, we see that ResNet-50 and VGG-16 did not have markedly different performance in learning the visual features for wildlife classification.

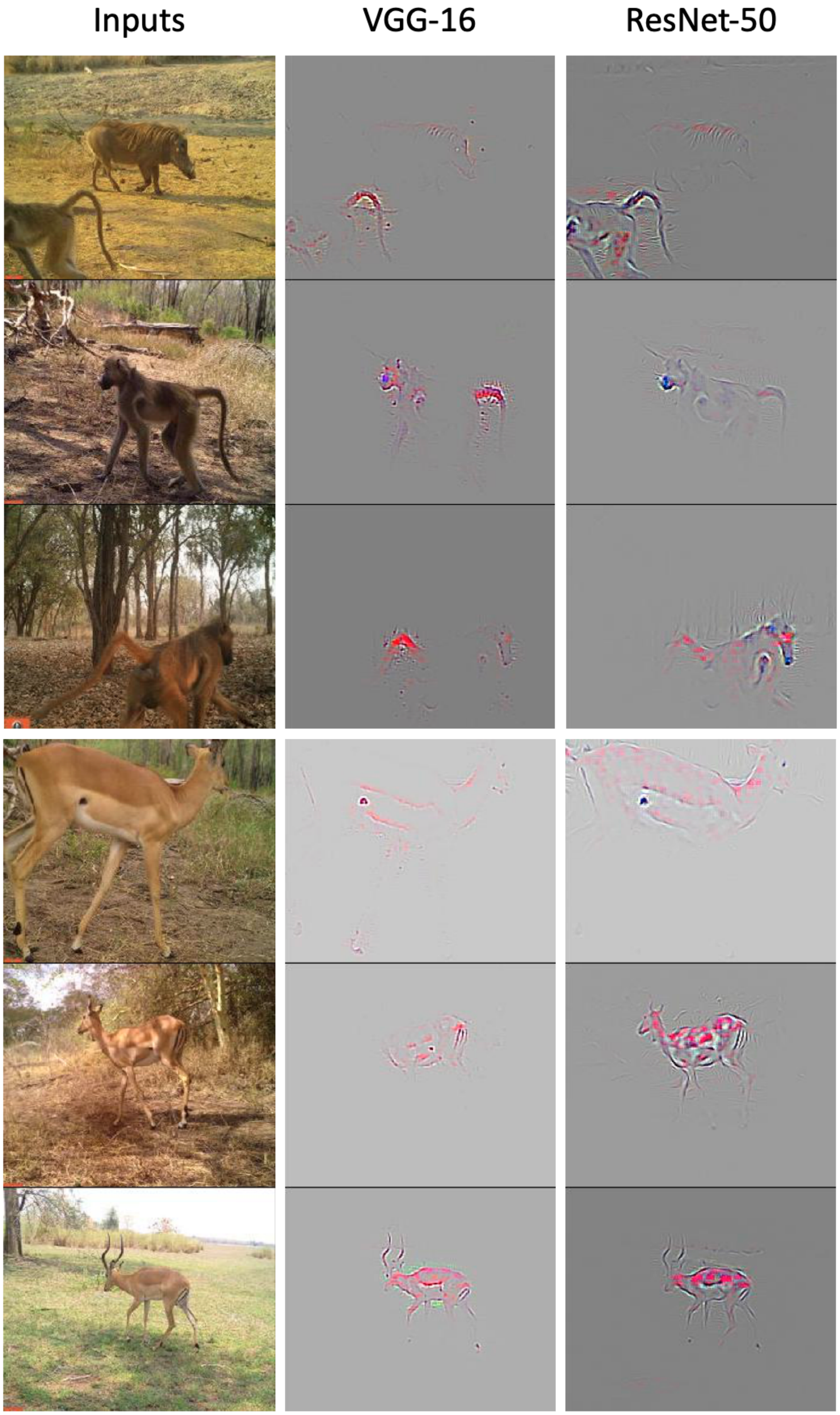

Figure 4. Localised feature comparison between VGG-16 and ResNet-50. Although both models extracted similar feature from the same images, ResNet-50 is more sensitive to the edges of target objects.

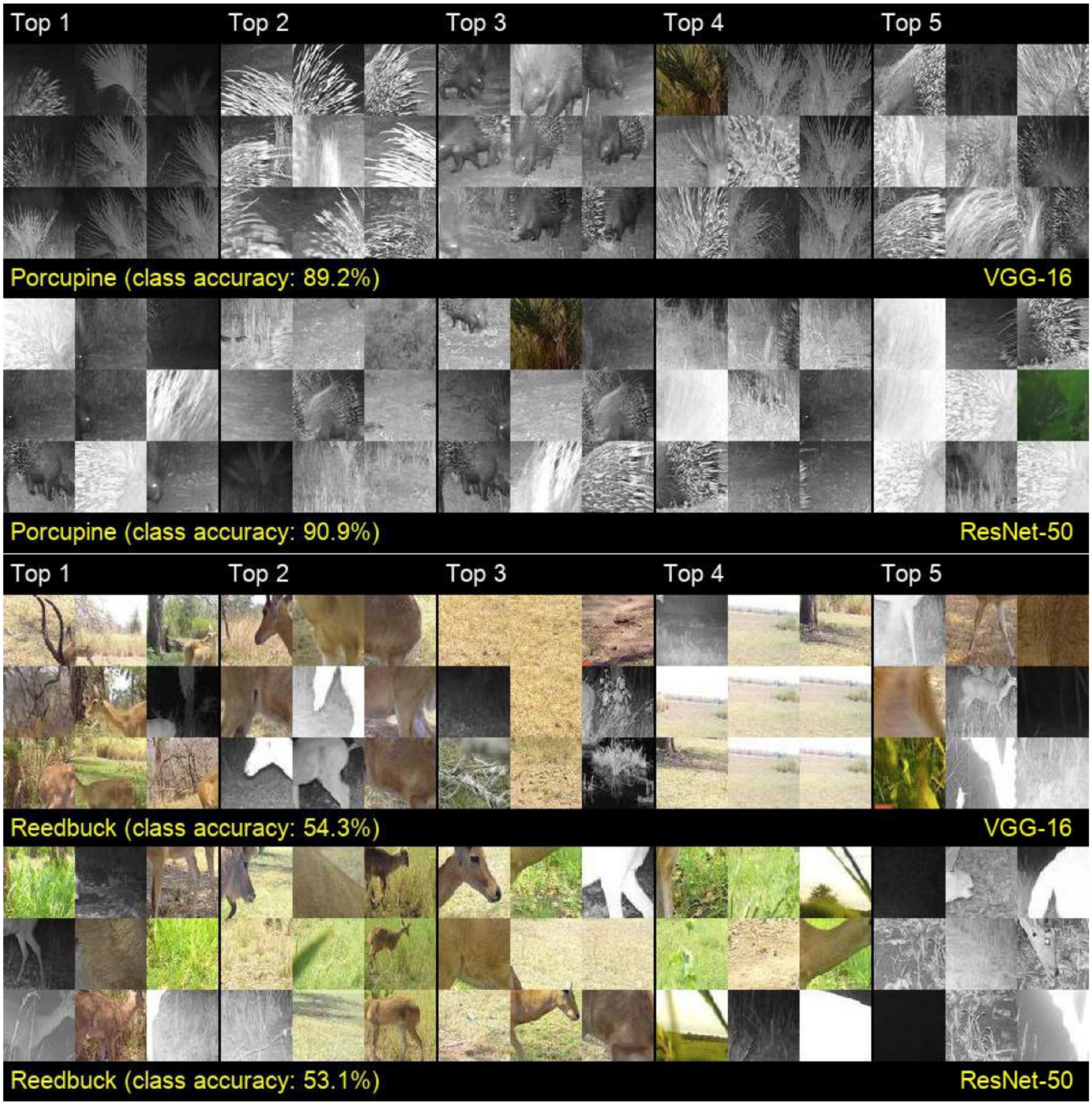

Figure 5. **Comparison of the MI results of VGG-16 and ResNet-50.** ResNet-50 was able to extract quills and heads of porcupines as well. For reedbuck images, ResNet-50 focused on relatively random elements from the images.

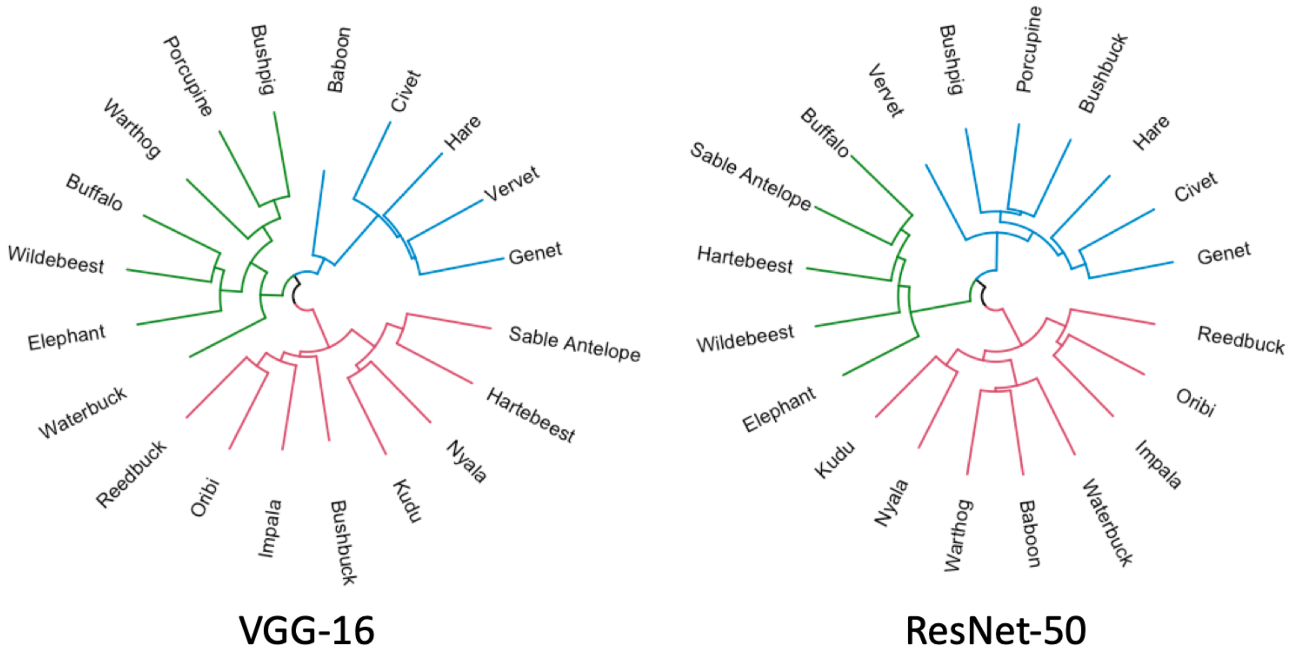

Figure 6. Comparison of hierarchical clustering results of VGG-16 and ResNet-50. The composition of the classes were somewhat different between the VGG-16 feature space and ResNet-50 feature space with the former identifying a more coherent "antelope" group (red branches) than the latter.

Localized feature of reedbuck

Here we present a more challenging example of localized feature extraction and human descriptor mapping. From Figure 7 we can see that the model used features that can hardly seen in most cases, such as the black spots under ears and black lines on the legs. This also increased the difficulties for the model to discriminate reedbucks from other animals.

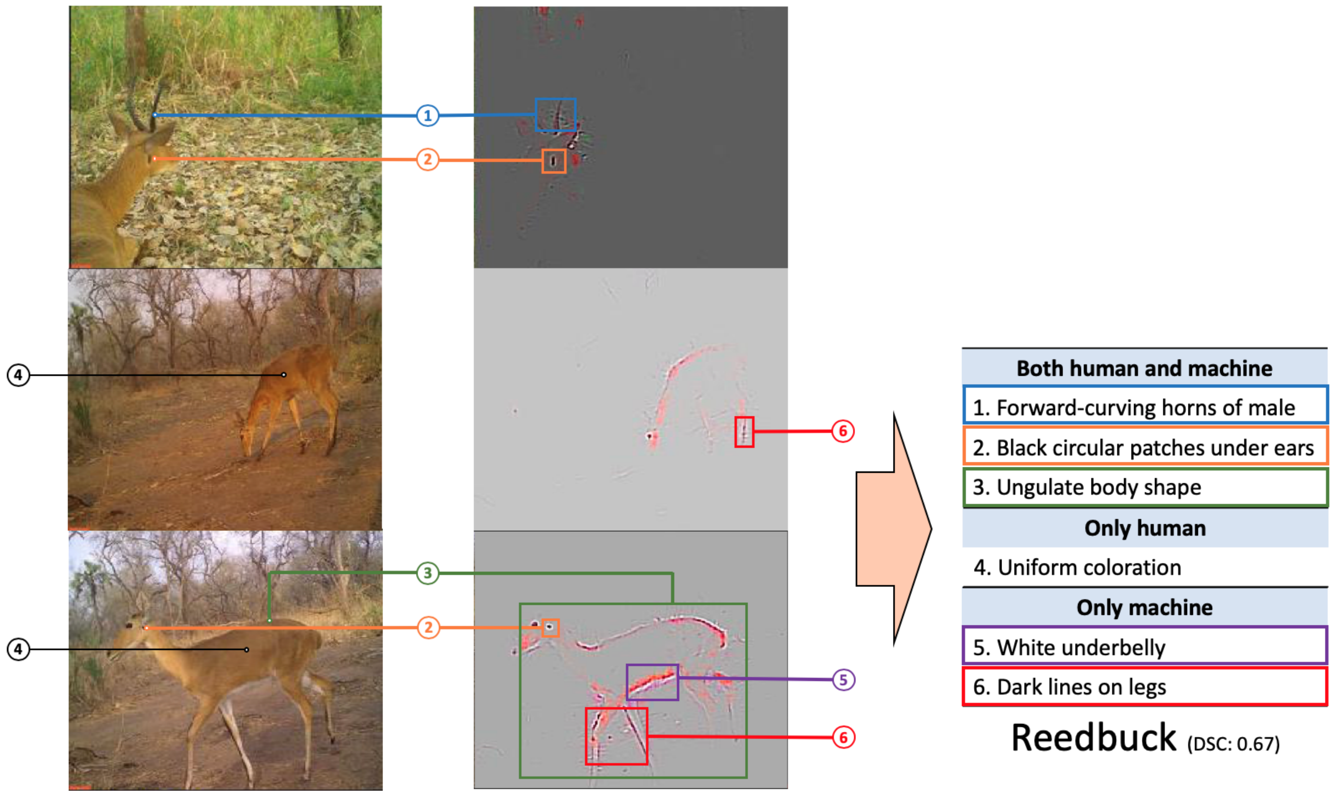

Figure 7. Localized features of reedbuck. The features of reedbucks were mostly obscure, and the extracted features (e.g. ungulate body shape, black circular patches under ears, white underbelly, and dark lines on legs) were not as discriminative compared with features of other species, such as the black stripes of impala, and white stripes of nyala. This might also be the reason why the class accuracy of reedbuck was not as good as other classes.

Extracted feature similarities

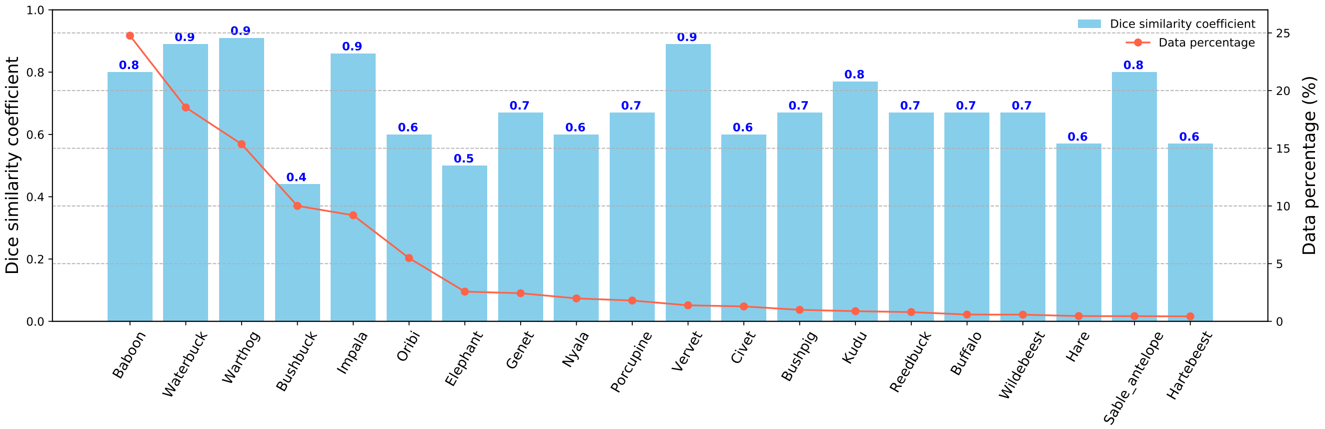

Figure 8. Similarities of extracted features that are to corresponding visual descriptors of each species created before these analyses started (Table 5). The extracted features were agreed upon by up to four authors (ZM, KMG, ZL and MSN), who scored 9 randomly selected images for each species. The similarities were calculated using Dice similarity coefficient. The scores seem to have no relationships with class distribution.

Mutual information results of all animal species

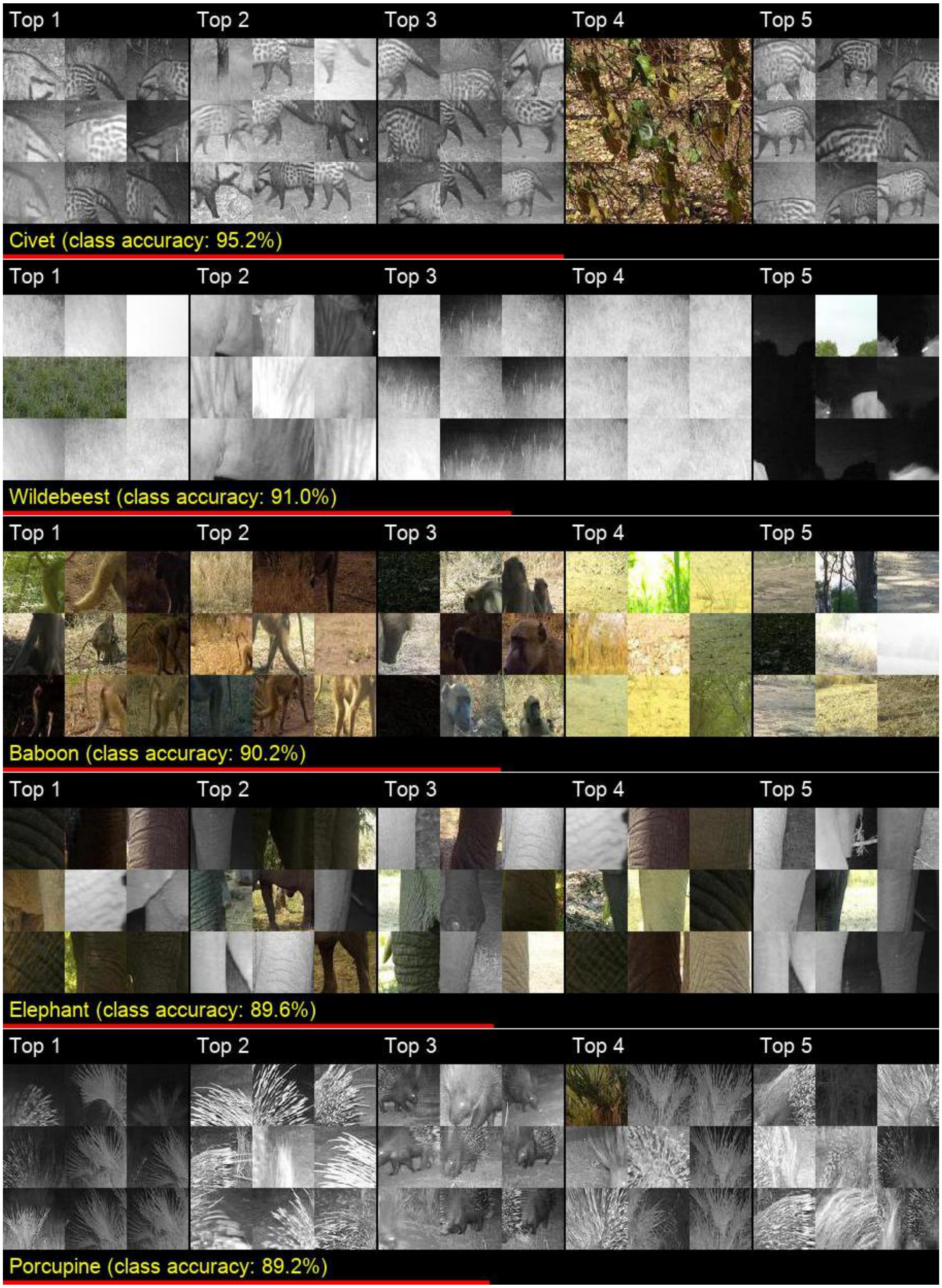

(a)

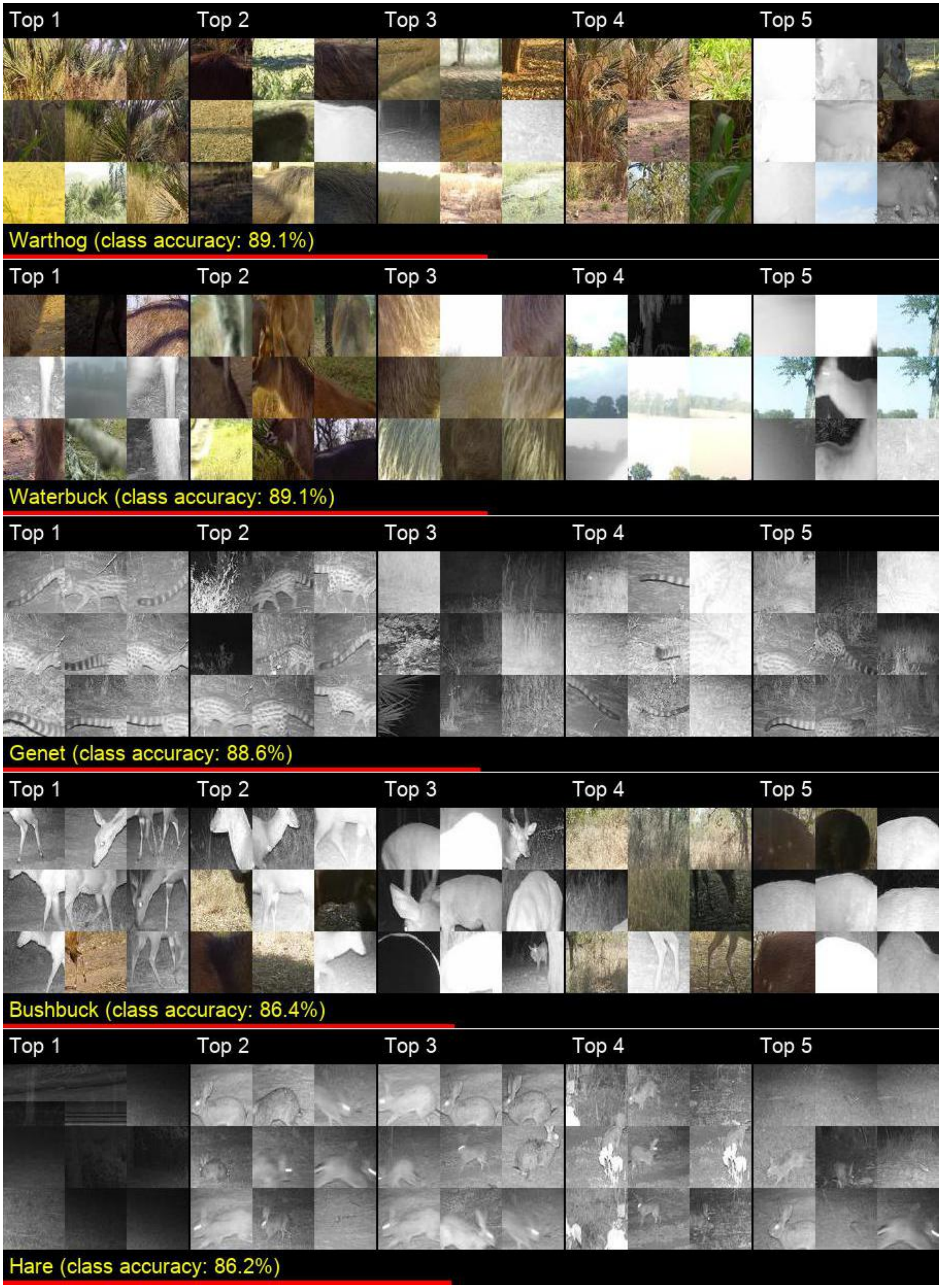
Top 1
Top 2
Top 3
Top 4
Top 5
Warthog (class accuracy: 89.1%)
Waterbuck (class accuracy: 89.1%)
Genet (class accuracy: 88.6%)
Bushbuck (class accuracy: 86.4%)
Hare (class accuracy: 86.2%)

(b)

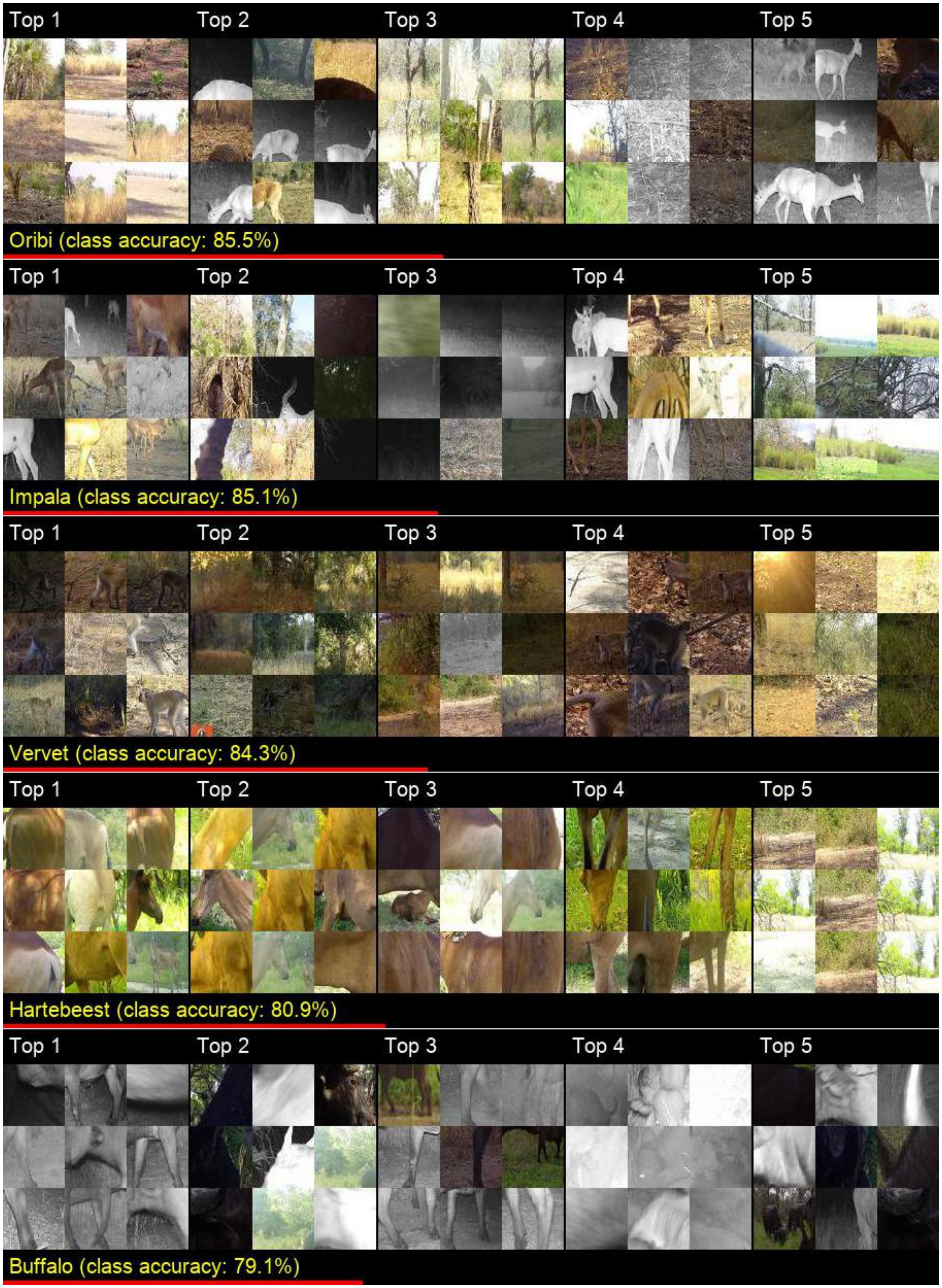
Top 1
Top 2
Top 3
Top 4
Top 5
Oribi (class accuracy: 85.5%)
Top 1
Top 2
Top 3
Top 4
Top 5
Impala (class accuracy: 85.1%)
Top 1
Top 2
Top 3
Top 4
Top 5
Vervet (class accuracy: 84.3%)
Top 1
Top 2
Top 3
Top 4
Top 5
Hartebeest (class accuracy: 80.9%)
Top 1
Top 2
Top 3
Top 4
Top 5
Buffalo (class accuracy: 79.1%)

(c)

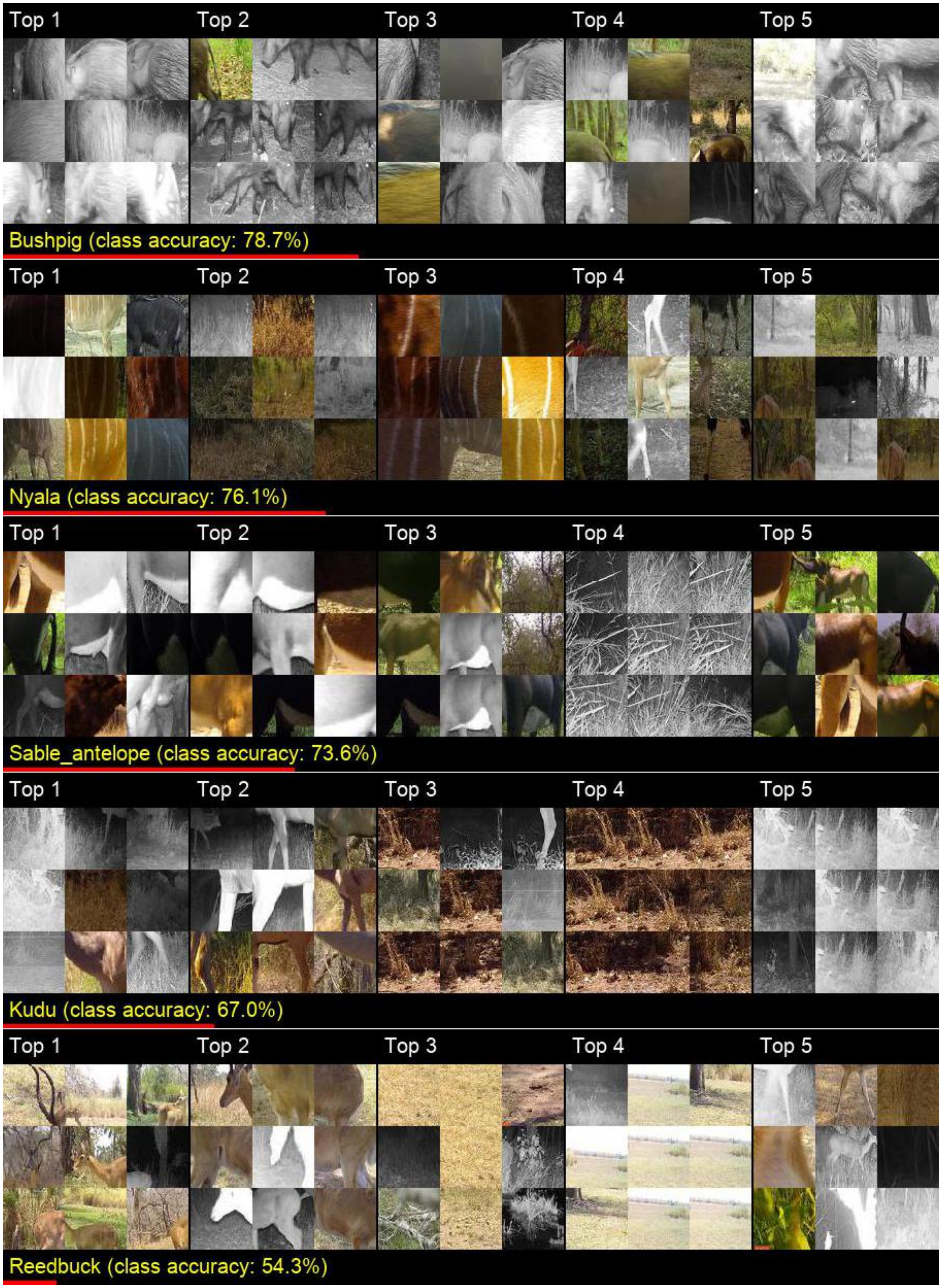

(d)

Figure 9. Following Figure 2, main text, for the case of porcupine and reedbuck, here the extracted patches are centered around the hottest pixel of the five most responsive neurons in the last convolutional layer of our CNN that has the highest MI score (Methods) for all 20 species (i.e., the first two are repeated for the sake of completeness). Red bars are graphical representations of class accuracy.

Appendix 6: Full visual descriptors

Table 5. Features used by humans to identify the 20 most common species from camera trap images. These features were identified through a survey of people with extensive experience in classifying camera trap data from Gorongosa. The features below were selected by at least 5 of the 13 survey respondents.

**Baboon**	primate body type
	tail curving upward at base
	long, dark snout
**Buffalo**	horns that curve to the side of head
	stocky barrel-shaped body
	dark coat
**Bushbuck**	thick ring of short, dark fur along neck
	parallel, slightly spiraled horns
	rounded rump
	ungulate body type
	white spots along the rump
**Bushpig**	pig body type
	silver-colored mane
**Civet**	nocturnal
	small carnivore body type
	black spots
	rounded back
	short, black legs
	crest of black hair from head to tail
**Elephant**	stocky, rectangular body shape
	gray to brownish wrinkled skin
	long trunk
	huge ears that are wide at base and narrow at bottom
	thick, round legs
	white tusks
**Genet**	slender body

	long, narrow tail<br>banded tail<br>black spots<br>small carnivore body type
**Hare**	round body<br>long ears that point up
**Hartebeest**	curved horns<br>ungulate body type<br>uniform dark brown coat
**Impala**	S-shaped horns of male<br>tri-colored body<br>black streaks on the rear
**Kudu**	long horns with large spirals (males)<br>hump on back of neck<br>thin, white stripes on back<br>white band between the eyes<br>light gray/brown color<br>ungulate body type<br>long, slender legs
**Nyala**	thin, white stripes on back<br>golden fur of female, dark brown fur of male<br>white spots on face and nose of male<br>spiral horns of male<br>ungulate body<br>white and yellow leg markings
**Oribi**	short, straight horns of male<br>white abdomen<br>short, black tail<br>black circular patches under ears<br>conical head shape<br>ungulate body type
**Porcupine**	nocturnal

	long black and white quills<br>stout, rounded body shape
**Reedbuck**	forward-curving horns of male<br>black circular patches under ears<br>uniform coloration<br>ungulate body shape
**Sable antelope**	long, backward-curving horns<br>horse-like body type<br>white striped facial markings<br>white underbelly<br>chestnut coat of female and dark brown color of male
**Vervet**	primate body type<br>black face<br>long tail, held out straight<br>white brow
**Warthog**	pig body type<br>two pairs of upward-pointing tusks<br>mane from top of head to middle of back<br>thin tail with tuft of hair at the bottom<br>flat, wide snout
**Waterbuck**	ribbed horns, curved out and forward (male)<br>white circular ring of fur on rump<br>shaggy, coarse, red-brown fur<br>black nose<br>ungulate body type
**Wildebeest**	curved horns that are wider than they are tall<br>horse-like body type<br>long, rectangular face<br>black beard<br>black mane along back<br>black vertical stripes on neck

References

1. Marcus Rowcliffe, J. Key frontiers in camera trapping research. *Remote. Sens. Ecol. Conserv.* **3**, 107–108, DOI: 10.1002/rse2.65 (2017).
2. Burton, A. C. *et al.* Review: Wildlife camera trapping: a review and recommendations for linking surveys to ecological processes. *J. Appl. Ecol.* **52**, 675–685, DOI: 10.1111/1365-2664.12432 (2015).
3. Steenweg, R. *et al.* Scaling-up camera traps: monitoring the planet's biodiversity with networks of remote sensors. *Front. Ecol. Environ.* **15**, 26–34, DOI: 10.1002/fee.1448 (2017).
4. Kays, R. *et al.* Does hunting or hiking affect wildlife communities in protected areas? *J. Appl. Ecol.* **54**, 242–252, DOI: 10.1111/1365-2664.12700 (2017).
5. Rich, L. N., Miller, D. A., Robinson, H. S., McNutt, J. W. & Kelly, M. J. Using camera trapping and hierarchical occupancy modelling to evaluate the spatial ecology of an african mammal community. *J. Appl. Ecol.* **53**, 1225–1235, DOI: 10.1111/1365-2664.12650 (2016).
6. Caravaggi, A. *et al.* A review of camera trapping for conservation behaviour research. *Remote. Sens. Ecol. Conserv.* **3**, 109–122, DOI: 10.1002/rse2.48 (2017).
7. LeCun, Y., Bengio, Y. & Hinton, G. E. Deep learning. *Nature* **521**, 436–444, DOI: 10.1038/nature14539 (2015).
8. Weinstein, B. G. A computer vision for animal ecology. *J. Animal Ecol.* **87**, 533–545, DOI: 10.1111/1365-2656.12780 (2017).
9. Chen, G., Han, T. X., He, Z., Kays, R. & Forrester, T. Deep convolutional neural network based species recognition for wild animal monitoring. In *2014 IEEE International Conference on Image Processing*, 858–862 (IEEE, 2014).
10. Norouzzadeh, M. S. *et al.* Automatically identifying, counting, and describing wild animals in camera-trap images with deep learning. *Proc. Natl. Acad. Sci.* DOI: 10.1073/pnas.1719367115 (2018). http://www.pnas.org/content/early/2018/06/04/1719367115.full.pdf.
11. Villa, A. G., Salazar, A. & Vargas, F. Towards automatic wild animal monitoring: Identification of animal species in camera-trap images using very deep convolutional neural networks. *Ecol. Informatics* **41**, 24–32, DOI: https://doi.org/10.1016/j.ecoinf.2017.07.004 (2017).
12. Siddiqui, S. A. *et al.* Automatic fish species classification in underwater videos: exploiting pre-trained deep neural network models to compensate for limited labelled data. *ICES J. Mar. Sci.* **75**, 374–389, DOI: 10.1093/icesjms/fsx109 (2018).
13. Sun, X. *et al.* Transferring deep knowledge for object recognition in low-quality underwater videos. *Neurocomputing* **275**, 897–908, DOI: https://doi.org/10.1016/j.neucom.2017.09.044 (2018).
14. Wäldchen, J. & Mäder, P. Machine learning for image based species identification. *Methods Ecol. Evol.* 1–10 (2018).
15. Yosinski, J., Clune, J., Bengio, Y. & Lipson, H. How transferable are features in deep neural networks? In *Proceedings of the 27th International Conference on Neural Information Processing System*, vol. 2, 3320–3328 (2014).
16. Swanson, A. *et al.* Snapshot serengeti, high-frequency annotated camera trap images of 40 mammalian species in an african savanna. *Sci. Data* **2**, 150026 EP – (2015).
17. Krizhevsky, A., Sutskever, I. & Hinton, G. E. Imagenet classification with deep convolutional neural networks. In Pereira, F., Burges, C. J. C., Bottou, L. & Weinberger, K. Q. (eds.) *Advances in Neural Information Processing Systems 25*, 1097–1105 (Curran Associates, Inc., 2012).
18. Simonyan, K. & Zisserman, A. Very deep convolutional networks for large-scale image recognition. *arXiv preprint arXiv:1409.1556* (2014).
19. He, K., Zhang, X., Ren, S. & Sun, J. Deep residual learning for image recognition. *2016 IEEE Conf. on Comput. Vis. Pattern Recognit. (CVPR)* 770–778, DOI: 10.1109/CVPR.2016.90 (2016).
20. Zeiler, M. D. & Fergus, R. Visualizing and understanding convolutional networks. In *Computer Vision – ECCV 2014*, 818–833 (Springer International Publishing, 2014).
21. Zhou, B., Khosla, A., Lapedriza, A., Oliva, A. & Torralba, A. Object detectors emerge in deep scene cnns. *arXiv preprint arXiv:1412.6856* (2014).
22. Liu, Z., Luo, P., Wang, X. & Tang, X. Deep learning face attributes in the wild. In *2015 IEEE International Conference on Computer Vision*, 3730–3738, DOI: 10.1109/ICCV.2015.425 (2015).

23. Selvaraju, R. R. *et al.* Grad-cam: visual explanations from deep networks via gradient-based localization. *2017 IEEE Int. Conf. on Comput. Vis.* 618–626, DOI: 10.1109/ICCV.2017.74 (2017).

24. Zhou, B., Bau, D., Oliva, A. & Torralba, A. Interpreting deep visual representations via network dissection. *IEEE Transactions on Pattern Analysis Mach. Intell.* 1–1, DOI: 10.1109/TPAMI.2018.2858759 (2018).

25. Lapuschkin, S. *et al.* Unmasking clever hans predictors and assessing what machines really learn. *Nat. Commun.* (2019).

26. Rajpurkar, P. *et al.* Deep learning for chest radiograph diagnosis: A retrospective comparison of the chexnext algorithm to practicing radiologists. *PLoS medicine* **15**, e1002686 (2018).

27. Seeland, M., Rzanny, M., Boho, D., Wäldchen, J. & Mäder, P. Image-based classification of plant genus and family for trained and untrained plant species. *BMC Bioinforma.* **20**, 4, DOI: 10.1186/s12859-018-2474-x (2019).

28. Wäldchen, J., Rzanny, M., Seeland, M. & Mäder, P. Automated plant species identification—trends and future directions. *PLOS Comput. Biol.* **14**, 1–19, DOI: 10.1371/journal.pcbi.1005993 (2018).

29. Shen, L., Lin, Z. & Huang, Q. Relay backpropagation for effective learning of deep convolutional neural networks. In *Computer Vision – ECCV 2016*, 467–482 (Springer International Publishing, Cham, 2016).

30. Paszke, A. *et al.* Automatic differentiation in pytorch (2017).

31. Deng, J. *et al.* Imagenet: A large-scale hierarchical image database. In *2009 IEEE Conference on Computer Vision and Pattern Recognition*, 248–255, DOI: 10.1109/CVPR.2009.5206848 (2009).

32. Van Horn, G. & Perona, P. The devil is in the tails: Fine-grained classification in the wild. *arXiv preprint arXiv:1709.01450* (2017).

33. Powers, D. Evaluation: From precision, recall and f-factor to roc, informedness, markedness & correlation. *Mach. Learn. Technol.* **2** (2008).

34. MacKay, D. J. C. *Information Theory, Inference & Learning Algorithms* (Cambridge University Press, 2002).

35. Battiti, R. Using mutual information for selecting features in supervised neural net learning. *IEEE Transactions on Neural Networks* **5**, 537–550, DOI: 10.1109/72.298224 (1994).

36. Peng, H., Long, F. & Ding, C. Feature selection based on mutual information criteria of max-dependency, max-relevance, and min-redundancy. *IEEE Transactions on pattern analysis machine intelligence* **27**, 1226–1238 (2005).

37. Murtagh, F. & Legendre, P. Ward's hierarchical agglomerative clustering method: which algorithms implement ward's criterion? *J. Classif.* **31**, 274–295, DOI: 10.1007/s00357-014-9161-z (2014).

38. He, K., Zhang, X., Ren, S. & Sun, J. Deep residual learning for image recognition. In *2016 IEEE Conference on Computer Vision and Pattern Recognition (CVPR)*, 770–778, DOI: 10.1109/CVPR.2016.90 (2016).
